# Fabrication of 3D Biomimetic Smooth Muscle Using Magnetic Induction and Bioprinting for Tissue Regeneration

**DOI:** 10.34133/bmr.0076

**Published:** 2024-09-09

**Authors:** Yang Luo, Zeming Hu, Renhao Ni, Rong Xu, Jianmin Zhao, Peipei Feng, Tong Zhu, Yaoqi Chen, Jie Yao, Yudong Yao, Lu Yang, Hua Zhang, Yabin Zhu

**Affiliations:** ^1^ Health Science Center, Ningbo University, Ningbo 315211, China.; ^2^ Sir Run Run Shaw Hospital, Zhejiang University School of Medicine, Hangzhou 310016, China.; ^3^ Ningbo Institute of Innovation for Combined Medicine and Engineering, The Affiliated Lihuili Hospital of Ningbo University, Ningbo 315046, China.; ^4^ The First Affiliated Hospital of Ningbo University, Ningbo 315010, China.; ^5^ Research Institute of Smart Medicine and Biological Engineering, Ningbo University, Ningbo 315211, China.; ^6^ State Key Laboratory of Molecular Engineering of Polymers, Fudan University, Shanghai 200438, China.

## Abstract

Smooth muscles play a vital role in peristalsis, tissue constriction, and relaxation but lack adequate self-repair capability for addressing extensive muscle defects. Engineering scaffolds have been broadly proposed to repair the muscle tissue. However, efforts to date have shown that those engineered scaffolds focus on cell alignment in 2-dimension (2D) and fail to direct muscle cells to align in 3D area, which is irresolvable to remodel the muscle architecture and restore the muscle functions like contraction and relaxation. Herein, we introduced an iron oxide (Fe_3_O_4_) filament-embedded gelatin (Gel)-silk fibroin composite hydrogel in which the oriented Fe_3_O_4_ self-assembled and functioned as micro/nanoscale geometric cues to induce cell alignment growth. The hydrogel scaffold can be designed to fabricate aligned or anisotropic muscle by combining embedded 3D bioprinting with magnetic induction to accommodate special architectures of muscular tissues in the body. Particularly, the bioprinted muscle-like matrices effectively promote the self-organization of smooth muscle cells (SMCs) and the directional differentiation of bone marrow mesenchymal stem cells (BMSCs) into SMCs. This biomimetic muscle accelerated tissue regeneration, enhancing intercellular connectivity within the muscular tissue, and the deposition of fibronectin and collagen I. This work provides a novel approach for constructing engineered biomimetic muscles, holding significant promise for clinical treatment of muscle-related diseases in the future.

## Introduction

Smooth muscle is extensively distributed in various tissues and organs throughout the human body. Smooth muscles rely on electromechanical coupling between muscle cells to facilitate contractile and relaxation functions, bearing essential physiological performances such as gastrointestinal peristalsis and blood flow regulation. Nevertheless, smooth muscle cells (SMCs), as terminally differentiated cells, exhibit limited proliferation and differentiation potential, imparting a weak self-repair capability to muscle tissues. This often proves insufficient to address circumferential or full-thickness muscle defects caused by trauma, tumors, or congenital diseases [1]. Currently, repairing such injuries with conventional clinical approaches, from pharmacological interventions to surgical procedures and other interventional strategies, frequently meets limited success. Moreover, cell therapies, for example, directly transplanting SMCs into the lesion, fail to support the original cellular arrangement, which is disastrous for intercellular signaling and the execution of contraction/relaxation functions [2–4]. Therefore, the in vitro construction of a biomimetic smooth muscle with in situ restoration and functional execution highlights an innovative treatment modality in regenerative medicine.

Previous research demonstrated that micropatterned engineering scaffolds could guide 2-dimensional (2D) alignment of muscle cells, assisting in smooth muscle regeneration [5] and providing insight into creating biomimetic muscle in vitro to replace or repair the diseased muscles. Furthermore, micropatterned SMC patches have also been extensively developed for tissue repair [6] and the aligned cell sheets can be procured by employing sacrificial engineering scaffolds or methods involving muscle delamination [7,8]. Williams et al. [9] successfully fabricated 3D tubular muscle by stacking multiple aligned cell sheets. However, these investigations concentrated solely on cell alignment in a 2D point through directional devices and failed to directly induce cells toward anisotropic 3D alignment of smooth muscle in vivo. Anisotropically aligned smooth muscle tissue orchestrates the spatiotemporal dynamics of excitation–contraction coupling, effectively modulating physiological functions. For example, the smooth muscle of the esophagus comprises an inner circular and an outer longitudinal layer. The 2 muscular layers asynchronously control the constriction and peristalsis of the esophagus, thereby efficiently propelling food from the pharynx to the stomach. Nonetheless, reconstructing anisotropically aligned smooth muscle tissue in vitro is challenging, necessitating self-organization at the cellular scale and macro-tissue/organ level.

The extracellular matrix (ECM) serves as critical topographical and biochemical cues during cellular growth [10,11]. It is also crucial for tissue development [12]. Due to the similarity to ECM in their interconnected hydrophilic network, hydrogels are often used as ECM substitutes for in situ loading cells, regulating cells’ proliferation, differentiation, and maturation. Researchers can shape hydrogels using techniques like lithography, electrospinning, and microfluidics [13–15]. However, replication of the intricate geometric structures of complex tissue/organs remains a considerable challenge.

The 3D bioprinting technology can precisely replicate the human tissue/organ architectures [16]. This allows accurate encapsulation of cells within the intricate structures of the biomimetic tissues/organs, providing technical support for the in vitro construction of biomimetic anisotropic muscle [17,18]. Traditional bioprinting methods require soft bioinks for effective cell encapsulation and vitality maintenance, which frequently results in poor integrity due to the rapid distortion and collapse of the intricate structures of the biomimetic tissues/organs [19]. In contrast, the embedded-based bioprinting system offers functional baths to collect the inks with appropriate fidelity and printability, effectively replicating the detailed structures of tissues/organs [20–22].

In our previous research, we introduced a novel *k*-carrageenan microgel suspension in the 3D bioprinting system, which offers high-precision and high-fidelity fabrication of complex tissue structures and high cell compatibility [23]. However, these efforts have afforded limited adjustment over the self-organization of anisotropic tissues. It can be attributed to the absence of micro/nanoscale geometric cues necessary for guiding 3D cellular alignment [24,25], and these geometric cues can also regulate cell differentiation [26]. Therefore, in this work, we innovatively engineered a hydrogel scaffold infused with aligned iron oxide (Fe_3_O_4_) stripes at the micro/nanoscale, which could induce cells to align along the micro/nanostripes in the 3D area.

Immune rejection is another issue faced by in vitro-constructed smooth muscle when transplanted allogeneically. Bone mesenchymal stem cells (BMSCs) have been proposed for low immunogenicity and high differentiation potential, making them suitable for allogeneic transplantations. Furthermore, research suggested that geometric cues can facilitate the differentiation of BMSCs into SMCs [25]. Accordingly, we integrated Fe_3_O_4_ stripes into the bioprinted hydrogel scaffold, enhancing the differentiation of BMSCs into SMCs. This bioprinting system holds significance for the self-organization of biomimetic smooth muscle.

**Fig. 1.**
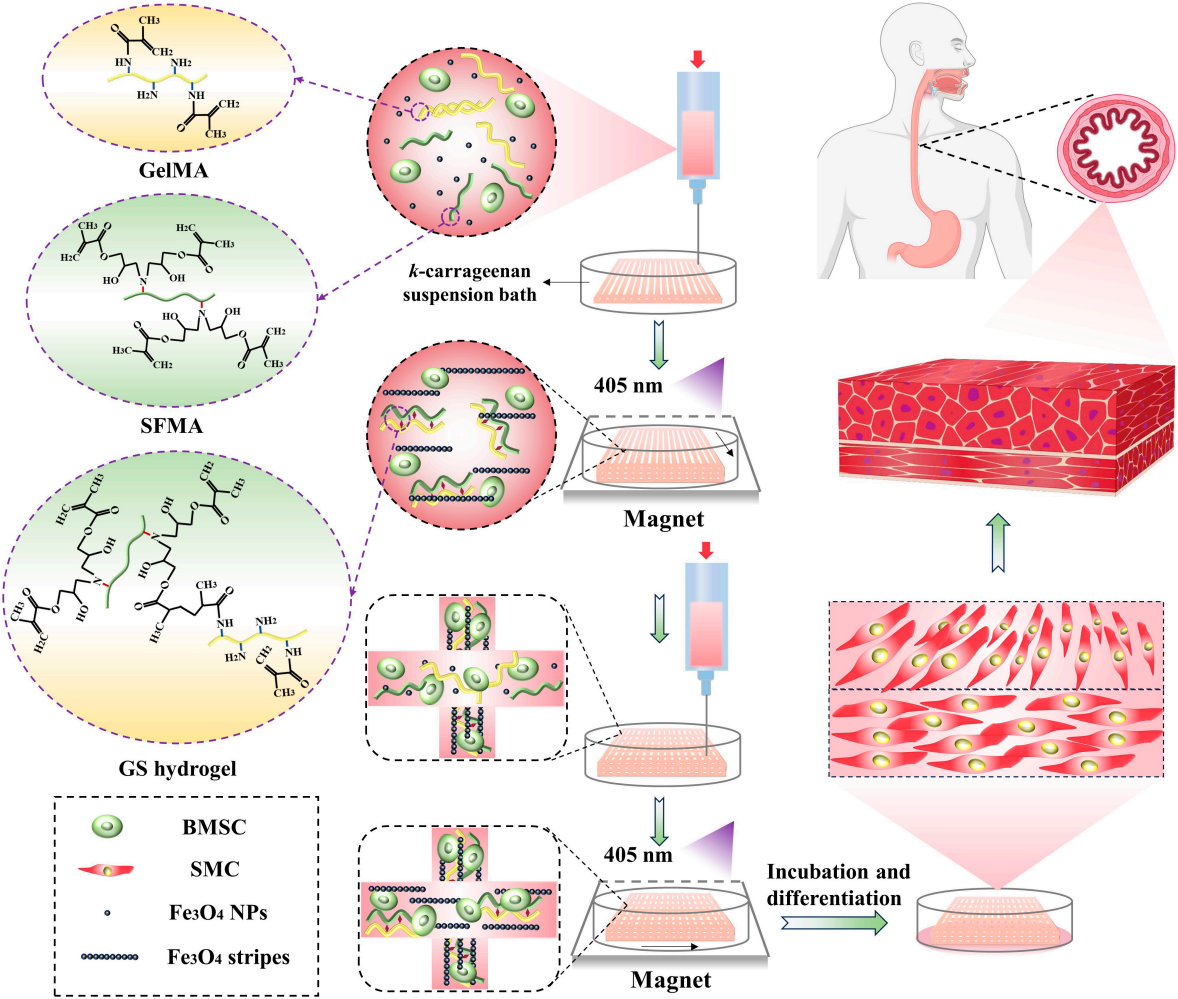
A schematic diagram of the biomimetic anisotropic smooth muscle constructed via 3D printing and magnetic induction technology.

In this study, we initially synthesized methacrylated gelatin (GelMA) and silk fibroin methacrylate (SFMA). The mechanical properties of the GelMA-SFMA composite hydrogel (GS hydrogel) were optimized by modulating the ratio of SFMA. 3D bioprinting technology was employed using the GS hydrogel as the matrix, combined with Fe_3_O_4_ nanoparticles (Fe_3_O_4_ NPs), induced to form the aligned stripes at micro/nanoscale under the induction of linear magnet force. This feature directed the SMCs to orient and grow in 3D area. Moreover, it could differentiate BMSCs into SMCs, thus enabling the construction of transplantable aligned or anisotropic muscle tissue. The workflow is displayed in Fig. 1. This biomimetic muscle promoted inner-circular and outer-longitudinal smooth muscle regeneration and self-organization in a rabbit model, repairing the defects on day 9 of transplantation. This work proposes a method for constructing biomimetic muscle, which holds significant implications for tissue regeneration.

## Materials and Methods

### Materials

Gelatin (Gel) and methacrylic anhydride (94% MA) were purchased from MACKLIN Biochemical Co. Ltd. (Shanghai, China). Moreover, *k*-carrageenan, Fe_3_O_4_ NPs (200 to 300 nm), and lithium phenyl(2,4,6-trimethylbenzoyl)phosphinate (LAP) were sourced from Aladdin Biochemical Technology Co. Ltd. (Shanghai, China). The reagent glycidyl methacrylate (GMA) was obtained from Sigma-Aldrich (Shanghai, China).

The immunological reagents included the following antibodies: anti-α-smooth muscle actin (α-SMA) rabbit antibody, anti-collagen I mouse antibody, anti-CD90 mouse antibody, and anti-CD34 rabbit antibody; secondary antibodies including goat anti-rabbit immunoglobulin G (IgG) conjugated to Alexa Fluor 488, goat anti-rabbit IgG linked to Alexa Fluor 594, goat anti-mouse IgG tagged with Alexa Fluor 488, and goat anti-mouse IgG associated with Alexa Fluor 594. These antibodies were all procured from Proteintech Group Inc., USA. Antibodies targeting connexin 43 (mouse) and fibronectin (rabbit) were acquired from Affinity Biosciences, USA. The actin-staining fluorophores tetramethyl rhodamine isothiocyanate (TRITC)-phalloidin and fluorescein isothiocyanate (FITC)-phalloidin were sourced from Solarbio Science & Technology Co. Ltd., Beijing, China.

### Synthesis of GelMA and SFMA

Gel (100 g) was dissolved in 1 l of phosphate-buffered saline (PBS) at 50 °C, followed by adding 20 ml of MA and stirred for 3 h at 50 °C. The mixed solution was dialyzed using deionized water for 5 d at 37 °C. The obtained GelMA was freeze-dried and stored at –80 °C for further use.

SFMA was synthesized according to the protocols in the literature [27,28]. Briefly, 40 g of sliced cocoons was added into Na_2_CO_3_ aqueous solution (2 l, 0.05 M) for 1 h at 100 °C to remove the sericin. The degummed silk fibroin was repeatedly cleaned with deionized water and dried at 50 °C for further weighing. Subsequently, degummed silk fibroin (25 g) was dissolved in 125 ml of lithium bromide (LiBr) aqueous solution (9.3 M) at 60 °C for 4 h. The mixed solution was filtered through gauze, dialyzed using deionized water for 5 d, and freeze-dried to obtain regenerated silk fibroin (RSF). Next, RSF (16 g) was dissolved in LiBr aqueous solution (9.3 M, 80 ml) again. GMA (4.8 ml) was added into the solution and stirred for 3 h at 60 °C to generate a reaction. Finally, the mixed solution was dialyzed using deionized water for 5 d and freeze-dried to obtain SFMA.

To determine the methacrylation, GelMA and SFMA were analyzed by ^1^H-NMR (nuclear magnetic resonance) on a Bruker NMR (400 MHz, German) with D_2_O as the solvent. Attenuated total reflection (ATR)-Fourier transform infrared (FTIR) (Nicolet 6700, Thermo Fisher Scientific, USA) was used to test the chemistry of GelMA and SFMA.

### Synthesis of GS hydrogels with varying polymeric compositions

GelMA (1 g) was dissolved in a 0.2% LAP aqueous solution (w/v, 5 ml) to produce a high-viscosity solution. Concomitantly, SFMA in various masses (0, 0.3, 0.6, 0.9, and 1.2 g) was also dissolved in a 0.2% LAP aqueous solution (w/v, 5 ml) to achieve SFMA solutions with various concentrations. Successively mixing GelMA and SFMA solutions in equal volumes facilitated the formulation of hydrogel precursors: 10% GelMA, 10% GelMA with 3% SFMA (G10S3), 10% GelMA with 6% SFMA (G10S6), 10% GelMA with 9% SFMA (G10S9), and 10% GelMA with 12% SFMA (G10S12). GS hydrogels with varying polymeric compositions were prepared after the solution was cured under ultraviolet (UV) light (405 nm).

### Assessment of rheological properties

Rheometric measurements were performed using a rheometer (HR-20, TA Instruments) to investigate the viscoelastic behavior of the UV-cured GS hydrogels. A time sweep experiment was performed to identify the cross-linking process. To ascertain the storage modulus (*G*′) and loss modulus (*G*″), parametric indicators of the elastic and viscous behaviors, a frequency sweep experiment was executed across a comprehensive spectrum ranging from 0.1 to 100 Hz. The tangent of the phase angle (tan δ) was used to display the ability of hydrogels to dissipate mechanical energy.

### Mechanical measurements

GS hydrogels were made into uniform cylindrical specimens and tested in a universal testing machine (CMT4000, SASCK) at a compressive speed of 5.0 mm/min. Young’s modulus was inferred through the initial slope of the stress–strain curve within a strain window extending from 15% to 25%. A cyclic compression test was carried out to demonstrate the deformation loss of hydrogels. The compression limit was set as 50% strain to protect hydrogels from cracking.

### Characterization of swelling kinetics and degradation behavior

Swelling kinetics of hydrogels (GelMA, G10S3, G10S6, G10S9, and G10S12) were evaluated. The hydrogels were made into uniform cylindrical specimens and weighed (*W*_0_) after being cured in UV light. Cylindrical hydrogels were immersed in PBS for 24 h at 37 °C to reach an equilibrium swelling. The weight of hydrogels was recorded (*W*_t_) at 0.5, 1, 2, 4, 8, and 24 h after immersion in PBS. The swelling ratio was calculated as the following formula: Swelling ratio (%) = (*W*_t_/*W*_0_ − 1) × 100%.

The degradation of hydrogel in vitro was analyzed using residual mass. The hydrogels were immersed in PBS and incubated at 37 °C for 28 d. At the time points 1, 3, 5, 7, 14, 21, and 28 d, the hydrogels were taken out and freeze-dried for 3 d. The mass of dried hydrogels was weighed (*W*_t_), and the initial weight of the sample after freeze-drying was recorded as *W*_0_. The Residual mass of hydrogels was calculated as the following formula: Residual mass (%) = *W*_t_/*W*_0_ × 100%.

### Microstructure measurements

The microstructure of hydrogels was observed using a scanning electron microscope (SEM; Phenom Pro). Freeze-dried hydrogels were torn apart and sputtered with gold using an ion sputter coater (ISC 150, SuPro Instrument). The microstructure of hydrogels was observed under SEM at 5 kV with 300× magnification. Pore diameter was measured and calculated using ImageJ software.

### Evaluation of hemocompatibility

Cylindrical hydrogels were incubated in diluted blood (10 times, with 0.9% NaCl solution) at 37 °C for 30 min. Deionized water-diluted blood and 0.9% NaCl solution-diluted blood were used as positive and negative controls, respectively. After incubation, hydrogels were washed with 0.9% NaCl solution and imaged to test the blood cell adhesion. The diluted blood was centrifuged at 750*g* for 5 min. The supernatants were collected, and the absorbance (*A*) was measured at 540 nm. Moreover, *A*_t_, *A*_PC_, and *A*_NC_ stand for the absorbances of hydrogels and positive and negative controls, respectively. The hemolysis ratio of hydrogels was calculated as the following formula: Hemolysis ratio (%) = (*A*_t_ − *A*_NC_)/(*A*_PC_ − *A*_NC_) × 100%.

### Cell viability and behaviors in hydrogels

Primary SMCs were extracted and cultured from rabbit esophagus using an adherent method and identified with anti-α-SMA rabbit antibody (1,800). Cells were cultured in the complete medium containing high-glucose Dulbecco’s modified Eagle’s medium (DMEM; VivaCell Biosciences) supplemented with 10% fetal bovine serum (FBS; VivaCell Biosciences) and 1% penicillin/streptomycin (NCM Biotech, China). Cells were mixed with GS hydrogel precursor solutions at a density of 1 × 10^7^ cells per ml and encapsulated in hydrogels using UV light. The viability of SMCs encapsulated in hydrogels was detected using a calcein-AM/PI (propidium iodide) staining kit (Solarbio, China). The fluorescence was observed under a confocal laser scanning microscope (CLSM; STELLARIS 5, Leica, Germany). The live cells were green, and the dead cells were red. Cell viability was calculated as the following formula: Cell viability (%) = Number of green/(Number of green + Number of red) × 100%.

SMCs were encapsulated in GS hydrogels with varying polymeric compositions and cultured in a humidified incubator at 37 °C with 5% CO_2_. The hydrogels were taken out on 3 and 5 d and fixed with 4% polyformaldehyde aqueous solution for 30 min. The encapsulated SMCs were penetrated with 0.5% Triton X-100 for 1 h, followed by incubation for 1 h with TRITC-phalloidin (1:300, Solarbio, China) at room temperature. The cells’ morphology was observed and imaged under CLSM.

### Printability of bioink

The G10S6 hydrogel precursor solution was selected as a bioink to test its printability. The supporting bath was 0.4% *k*-carrageenan. Specifically, the *k*-carrageenan was dissolved in PBS and broken into a liquid form using a blender after it formed a hydrogel. One syringe loaded with G10S6 hydrogel precursor solution was used to print organ-mimicked shapes. Macroscopic shapes and microscopic structures were imaged using a camera and CLSM, respectively.

G10S6 hydrogel precursor solution loaded with SMCs was printed into the *k*-carrageenan bath to obtain a lattice-shaped scaffold, followed by cross-linking under UV light for 2 min. The scaffold was taken from the bath and cultured in a DMEM complete medium. The 3D bioprinting parameters were as follows: (a) The density of SMCs was 2 × 10^7^ cells per ml; (b) the diameter of the nozzle was 210 μm; (c) the velocity of extrusion was 0.1 ml/min; (d) the velocity of print was 20 mm/s.

The viability of the encapsulated SMCs was detected using a calcein-AM/PI staining kit followed by 24 h of culturing. The cytoskeleton was stained with FITC-phalloidin (1:300, Solarbio, China) after culturing for 48 h. The cell morphology was observed and imaged using CLSM.

### 3D-printed scaffold for SMC orientation and self-organization

Furthermore, a 3D-printed scaffold with parallel-arranged bundles was designed for SMC orientation and self-organization. Magnetic Fe_3_O_4_ NPs were introduced and transformed into Fe_3_O_4_ stripes under a magnetic field, acting as topographical and biochemical cues to guide cellular orientation and growth. Specifically, G10S6 hydrogel precursor solution, magnetic Fe_3_O_4_ NPs (0.2%, w/v), SMCs, and LAP (0.2%, w/v) were mixed to act as a bioink. Bioink without magnetic Fe_3_O_4_ NPs were used as the control. The 3D bioprinting parameters were the same as mentioned in the “Printability of bioink” section. The cell-containing scaffold was positioned within a magnetic field (aligned with the direction of the bundles within the scaffold) and cross-linked under UV light (405 nm) for 2 min. This resulted in the formation of a cellular scaffold with Fe_3_O_4_ stripes. Then, the scaffold was incubated in a DMEM complete medium for 9 d. At 3, 6, and 9 d, SMCs were stained with FITC-phalloidin and observed under CLSM.

### The fabrication of transplantable aligned smooth muscle

BMSCs were extracted from the bone marrow of rabbits by adherent method and cultured in a Roswell Park Memorial Institute 1640 medium (RPMI1640, VivaCell Biosciences) supplemented with 10% FBS (VivaCell Biosciences) and 1% penicillin/streptomycin (NCM Biotech, China). The nature of BMSCs was confirmed by detecting the expression of the positive (CD90) and negative markers (CD34). The BMSCs were induced into chondroblasts, adipocytes, and osteoblasts using the differentiation kit (Procell, China) to verify differentiation ability.

BMSCs were encapsulated in the hydrogel scaffold with parallel-arranged bundles. To differentiate BMSCs into SMCs, the scaffold was first cultured in RPMI1640 complete medium for 3 d. Next, transforming growth factor-β1 (TGF-β1) (10 ng/ml) was added to the complete medium and kept for another 3 d. The medium was changed into SMCs’ special medium (icell, China), supplemented with TGF-β1 (10 ng/ml), and cultured for 3 d. After that, this culture was maintained in SMCs’ special medium.

Cellular morphology and CD90 expression, a positive marker of BMSCs, were evaluated in BMSCs after cultivation for 3 d within the muscle scaffold. α-SMA is a specific marker for SMCs. On 6, 9, and 12 d of incubation, the expression of α-SMA in the cells was tracked to assess the differentiation of BMSCs into SMCs.

### The fabrication of anisotropic smooth muscle

Anisotropic smooth muscle was fabricated using the 3D bioprinting system. The components of BMSC-loaded bioink and the printing parameters were the same as in the “3D-printed scaffold for SMC orientation and self-organization” section. The bioink was printed to obtain an align-patterned scaffold, which was further cross-linked under UV light. Subsequently, the same bioink was printed onto the cross-linked scaffold using a similar method but in a perpendicular direction. After UV cross-linking and magnet-induced orientation, the multi-hierarchical scaffold was treated following the method described in the “The fabrication of transplantable aligned smooth muscle” section. Finally, the smooth muscle with multi-hierarchical configurations was obtained. The cytoskeleton of the muscle was stained with FITC-phalloidin and observed under CLSM to explore the muscular features.

### Evaluation of smooth muscle regeneration

Before conducting smooth muscle repair experiments, we evaluated the biocompatibility and degradation of hydrogel G10S6 containing Fe_3_O_4_ NPs in vivo. Four New Zealand rabbits (2 months old) were used in this study. Rabbits were anesthetized with isoflurane using anesthetic delivery equipment (R510-29, RWD LIFE Science Co. Ltd). The gas flow rate of isoflurane was set at 3 l/min before anesthesia and adjusted to 2 l/min during the operation. Moreover, O_2_ was maintained at 1 to 2 l/min during the experiment. G10S6 hydrogel precursor solution (200 μl) containing Fe_3_O_4_ NPs (0.2%, w/v) was injected subcutaneously into rabbits and crosslinked using UV light for 2 min. Postoperative evaluations were conducted at 3, 6, and 9 d. The corresponding skin tissue was collected and histologically examined.

New Zealand rabbits were divided into 2 groups (control group and treatment group, *n* = 9). The muscle tissue of the rabbit esophagus was defective (~3 × 5 mm in size) after the animals were anesthetized with isoflurane. The fabricated muscle was employed to replace the defective muscle. The tissue was seamed with the biological glue (Jitian Bio, China), and the skin was sealed with the sutures. The control animal was processed consistently; however, no muscle replacement was performed. Postoperative evaluations were conducted at 3, 6, and 9 d. The corresponding esophagus tissue was collected and histologically examined.

The animal experiment protocol was reviewed and approved by the Animal Ethics and Welfare Committee of Ningbo University. The animals used in the experiments were housed in an environment with a constant temperature of 25 °C and humidity of 60%, provided with ample water and food. Before collecting tissue samples, the animals were euthanized using an overdose of isoflurane. Institutional animal ethics approval was taken prior to animal experiments (approval number: NBU20220159).

### Histological analysis

To evaluate the biocompatibility and degradation of hydrogel G10S6 containing Fe_3_O_4_ NPs, the skin tissue was cut into slices (~6 μm in thickness) on a frozen microtome (CryoStar NX50, Thermo Fisher Scientific, USA) and stained with hematoxylin & eosin (H&E) kit according to the manufacturer’s instructions. The morphology was observed under an optical microscope (Nexcope, NIB410, China).

The esophagus tissue underwent the same treatment to evaluate the integrity of esophageal muscle repair. Furthermore, we performed F-actin staining on muscle tissues and observed the arrangement of SMCs under a CLSM.

Immunofluorescent (IF) staining was done to detect the expression of α-SMA, collagen I, connexin 43, and fibronectin, proteins that are closely associated with the contractile function of smooth muscle tissue and the production of ECM. The tissue slices were penetrated and sealed with Triton X-100 (0.2%) and bovine serum albumin (3%, Solarbio Life Sciences, China) for 1 h. It was followed by incubation with the anti-α-SMA rabbit antibody (1:800), anti-collagen I mouse antibody (1:300), anti-connexin 43 mouse antibody (1:300), and anti-fibronectin rabbit antibody (1:300) for 2 h at room temperature. Then, incubation was conducted for another 1 h with the goat anti-rabbit IgG (1:500, Alexa Fluor 488) and the goat anti-mouse IgG (1:500, Alexa Fluor 594). TRITC-phalloidin was used to stain the cytoskeleton, and 4′,6-diamidino-2-phenylindole (DAPI) was used to stain the nucleus. The tissue slices were observed using CLSM.

### Statistical analysis

Data were presented as mean ± standard deviation (SD). The assessment of statistical significance was conducted using Student’s unpaired *t* test and one-way analysis of variance (ANOVA). *P* < 0.05 was considered statistically significant.

## Results

### Synthesis of GelMA and SFMA

GelMA and SFMA were matrix materials of GS hydrogel. The synthesis route for GelMA and SFMA is provided in Figs. S1A and S2A. ^1^H-NMR and ATR-FTIR spectra of GelMA and SFMA were taken to characterize their composites. Figure S1B illustrates 2 more peaks at 5.48 and 5.72 parts per million (ppm) for GelMA compared to Gel in ^1^H-NMR spectra. This indicated that the methacrylate substitution on Gel succeeded. The result of the ATR-FTIR measurement revealed the enhancement of the vibrational peaks of C–NH–C (3,450 cm^−1^) and C═C (1,640 cm^−1^), suggesting that the GelMA was synthesized successfully (Fig. S1C). Similarly, SFMA was synthesized by methacrylate substitution of the RSF. The ^1^H-NMR spectra of SFMA displayed 2 peaks (5.90 and 6.32 ppm), which were not exhibited in RSF molecules (Fig. S2B). The ATR-FTIR spectra in SFMA and RSF samples illustrated peaks of amide I, II, and III at 1,637, 1,515, and 1,238 cm^−1^, respectively. However, a small shift was observed in SFMA at 952 and 1,168 cm^−1^, representing the CH_2_ wagging stretching of the methacrylate vinyl group in GMA (Fig. S2C). The MA and GMA functional groups were only slightly detectable or hidden as the molecular weight of MA and GMA was much smaller than that of the Gel and RSF, respectively. These results confirmed that GelMA and SFMA were synthesized successfully.

### Composition optimization of GS hydrogel

The biomimetic scaffolds must possess appropriate biomechanics to adapt to the tissue that will be replaced. GelMA, as a traditional and exemplary bioink, possesses excellent biocompatibility, allowing rapid pseudopodia extension for the cells embedded within bioinks [29,30]. However, the brittleness of GelMA makes it unsuitable as a replacement material for muscle requiring contractile and relaxational functionality [31]. SFMA, as a protein-based polymer material, exhibits good biocompatibility and high mechanical strength [32,33]. Incorporating SFMA could potentially compensate for the poor mechanical properties and strength of GelMA. Thus, the GS hydrogel recoverability upon large deformation is maintained to meet the contractile and relaxational functionality of muscle tissue. However, while improving the strength, it might be difficult for the GS hydrogels to degrade and restrict the rapid pseudopodia extension of cells embedded within them. Therefore, the compositional optimization of the GS hydrogel is critical to ensure the properties of recoverability and strength.

**Fig. 2.**
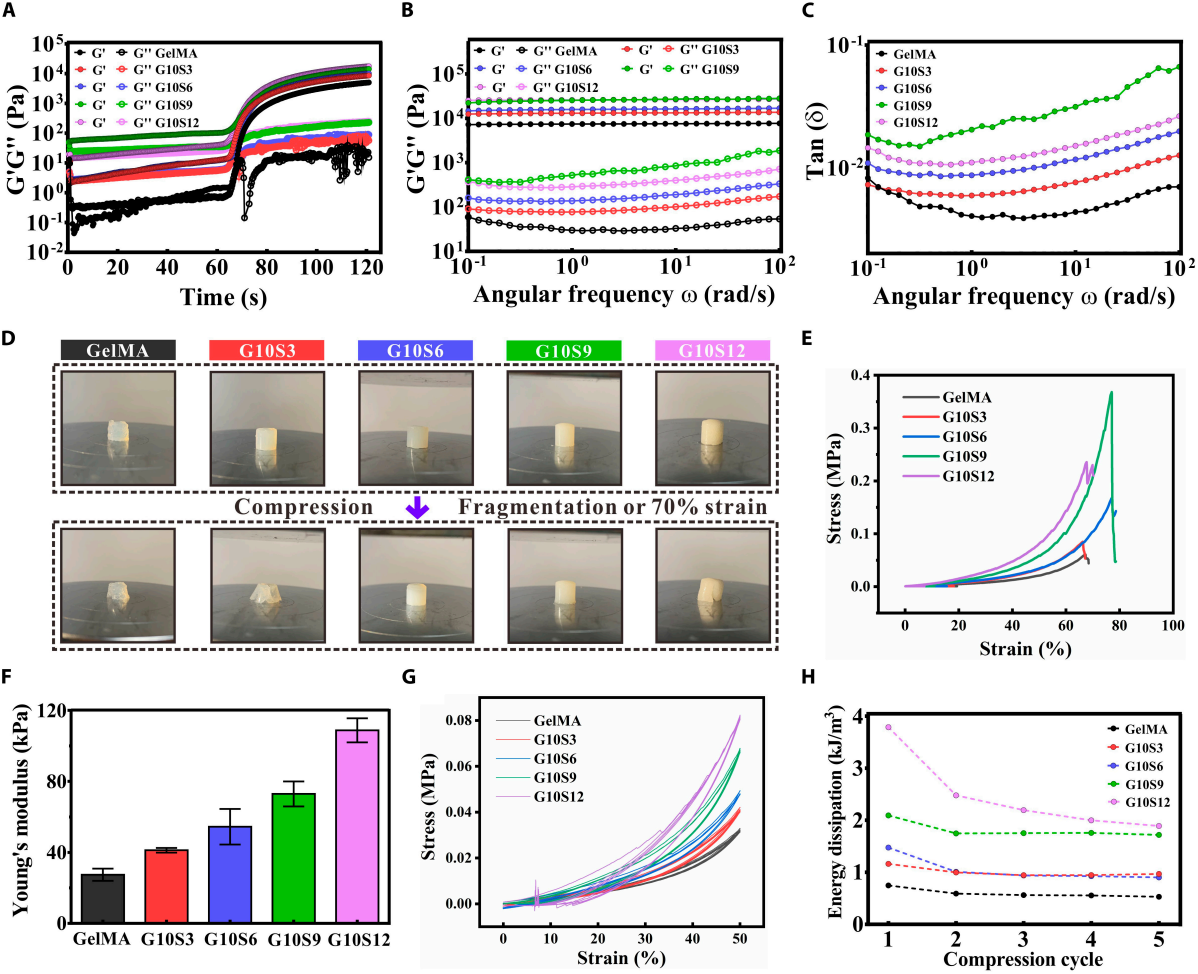
Rheological and mechanical properties of GS hydrogel. (A) Gelation kinetics with exposure to UV light. (B) Dependence of storage modulus (*G*′) and loss modulus (*G*″) on the frequency sweep after hydrogels were cured under UV light. (C) Dependence of tan (δ) on the sweep frequency. (D) Photographs illustrating the different modes of compressive failure in a mechanical test among the hydrogels following compression to 70% strain: brittle (GelMA, G10S3, and G10S12) and recoverable (G10S6 and G10S9). (E) Compressive stress–strain curves and (F) Young’s modulus of the hydrogels. (G) Dependence of stress on the continuous strain. (H) Energy dissipation of hydrogels in every compression cycle.

Five groups of hydrogel precursor solutions were prepared for rheological measurements. As displayed in Fig. 2A, *G*′ was much higher than *G*″ following photo-crosslinking for 30 s, indicating that the precursor solutions transformed into a gel state. The frequency sweep revealed that 5 groups of GS hydrogel were relatively stable because *G*′ (representing the elastic property) did not change greatly under different shear modes. *G*″, demonstrating the viscous property, increased slightly with the increase of angular frequency (Fig. 2B). Tan (δ) reflected the energy dissipation property of GS hydrogel under shear modes. The results found that GS hydrogels demonstrated higher energy dissipation as the concentration of SFMA in GS hydrogel increased. Nevertheless, hydrogel G10S9 possessed higher energy dissipation than hydrogel G10S12 (Fig. 2C), which might be induced by the excessive SFMA in the hydrogel.

Upon compression measurement, the pure GelMA hydrogel and hydrogels G10S3 and G10S12 exhibited rigidity and suddenly failed at 60% to 70% strain. However, hydrogels G10S6 and G10S9 displayed excellent deformability with no rupture until 70%, as displayed in Fig. 2D and E. In addition, as the concentration of SFMA in GS hydrogel increased, Young’s modulus of GS hydrogel increased (Fig. 2F). The results revealed that the elastic properties of GS hydrogel were proportional to the concentration of SFMA components under the stress modes. A cyclic compression test was conducted to assess the deformation loss of GS hydrogels (Fig. 2G). The deformation loss of each GS hydrogel decreased as the compression cycle increased. Hydrogel G10S12 demonstrated the maximum deformation loss, while GelMA hydrogel exhibited the minimum deformation loss in each compression cycle (Fig. 2H).

These results verified that the incorporation of SFMA into the GelMA substrate substantively augmented the mechanical properties of the hydrogels. Particularly, hydrogels G10S6 and G10S9 displayed exceptional pliability and could revert to their original structure without fracture, even when subjected to a formidable 70% strain. This attribute is of paramount importance for materials used as muscular tissue substitutes. Indeed, it was imperative to investigate the impacts of the hydrogel microenvironment on cellular behaviors.

**Fig. 3.**
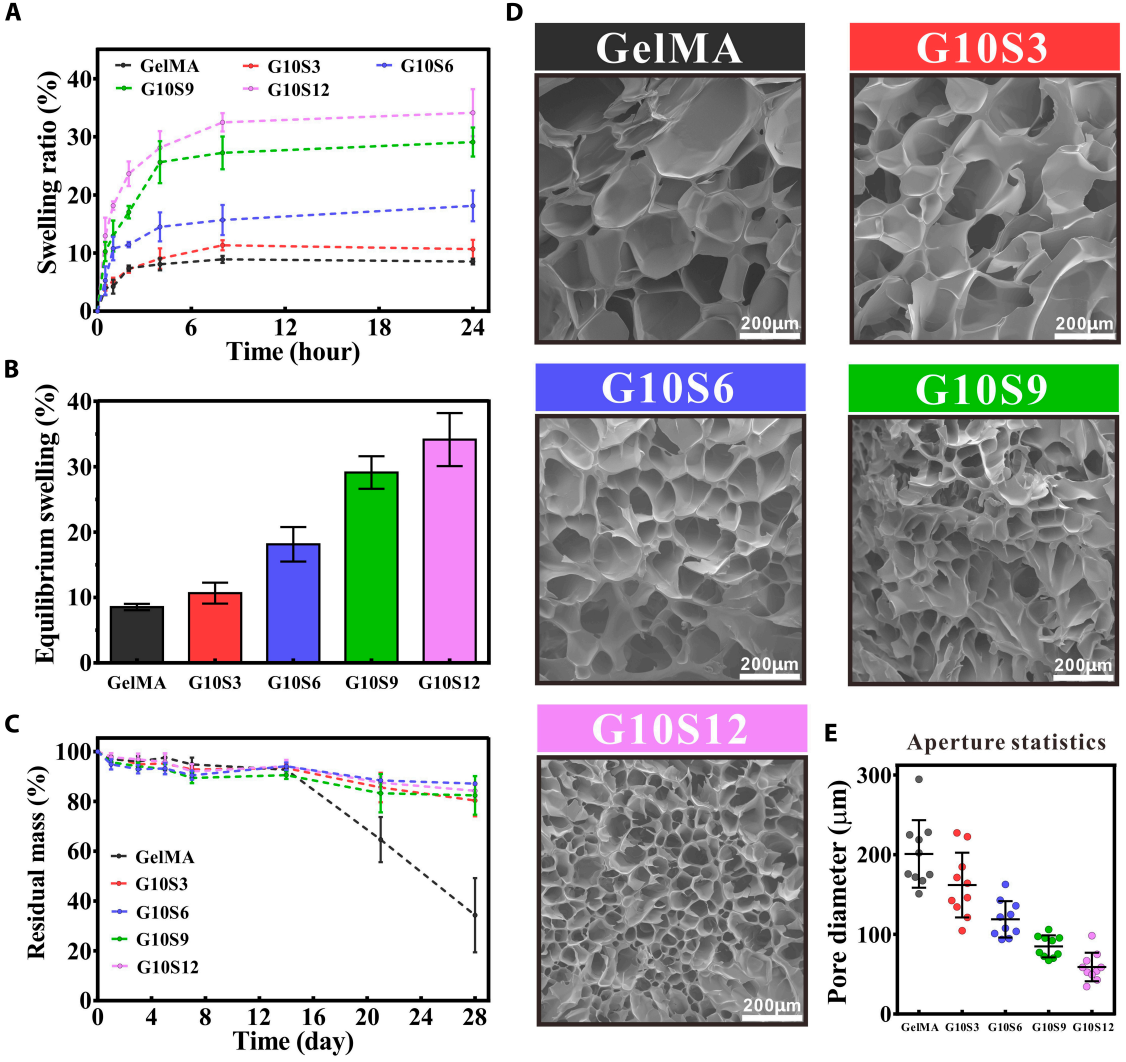
Swelling kinetics, degradation profiles, and microstructural properties of 5 GS hydrogels. (A) Swelling curves. (B) Equilibrium swelling ratio. (C) Degradation behaviors in PBS. (D) SEM observation. (E) Pore diameter.

In cellular 3D printing applications, the swelling behavior is critical since it directly relates to the culture medium penetration and diffusion. As illustrated in Fig. 3A, PBS permeated all GS hydrogels for the first 8 h and slowly reached a state of balance. The equilibrium swelling rate was maximum in hydrogel G10S12 (34.14 ± 4.057%) and minimum (8.542 ± 0.4878%) in GelMA hydrogel (Fig. 3B).

The degradation behavior and pore size of hydrogels were related to the spreading behavior of cells inside the hydrogels. The results demonstrated that GelMA hydrogel degraded faster to a greater extent than all GS hydrogels during the test time (Fig. 3C). Notably, the weight of each GS hydrogel decreased very little, with few differences between them. This could be because the introduction of SFMA densified the cross-linked network, making it difficult to degrade during the test time. The explanation was further confirmed by pore size analysis, which was inversely proportional to the concentration of SFMA in hydrogels; the more SFMA component, the denser cross-linked network (smaller pore size; Fig. 3D and E).

GS hydrogel could be used as the scaffold matrix due to its good blood compatibility (Fig. S3) and cell compatibility (Fig. S4; the esophageal SMCs were used as model cells). Furthermore, the behaviors of SMCs encapsulated in hydrogels were investigated. The results revealed that SMCs could grow in all GS hydrogels; however, they exhibited slower pseudopodia extension than in pure GelMA hydrogel. Meanwhile, with the increased SFMA concentration in the GS hydrogel, the pseudopodia extension rate decreased in the cells. The cells did not spread in the G10S12 hydrogel with the highest SFMA concentration due to the compactness and the smallest pore size (Fig. S5).

The biomimetic scaffold must possess good mechanical properties to meet the muscle contractile and relaxational functionality. The internal environment must be suitable for the survival, spreading, and directional arrangement of SMCs. The covalently cross-linked G10S6 hydrogel was developed for cell-adaptable matrices. The resulting product demonstrated high mechanical stiffness reaching 54.45 kPa and dynamic degradability, cell viability up to 91.35%, and fast morphogenesis within 3 d. Accordingly, hydrogel G10S6 was optimized according to the requirements of muscle biomimetic scaffolds.

### Printability of G10S6 hydrogel precursor solution

**Fig. 4.**
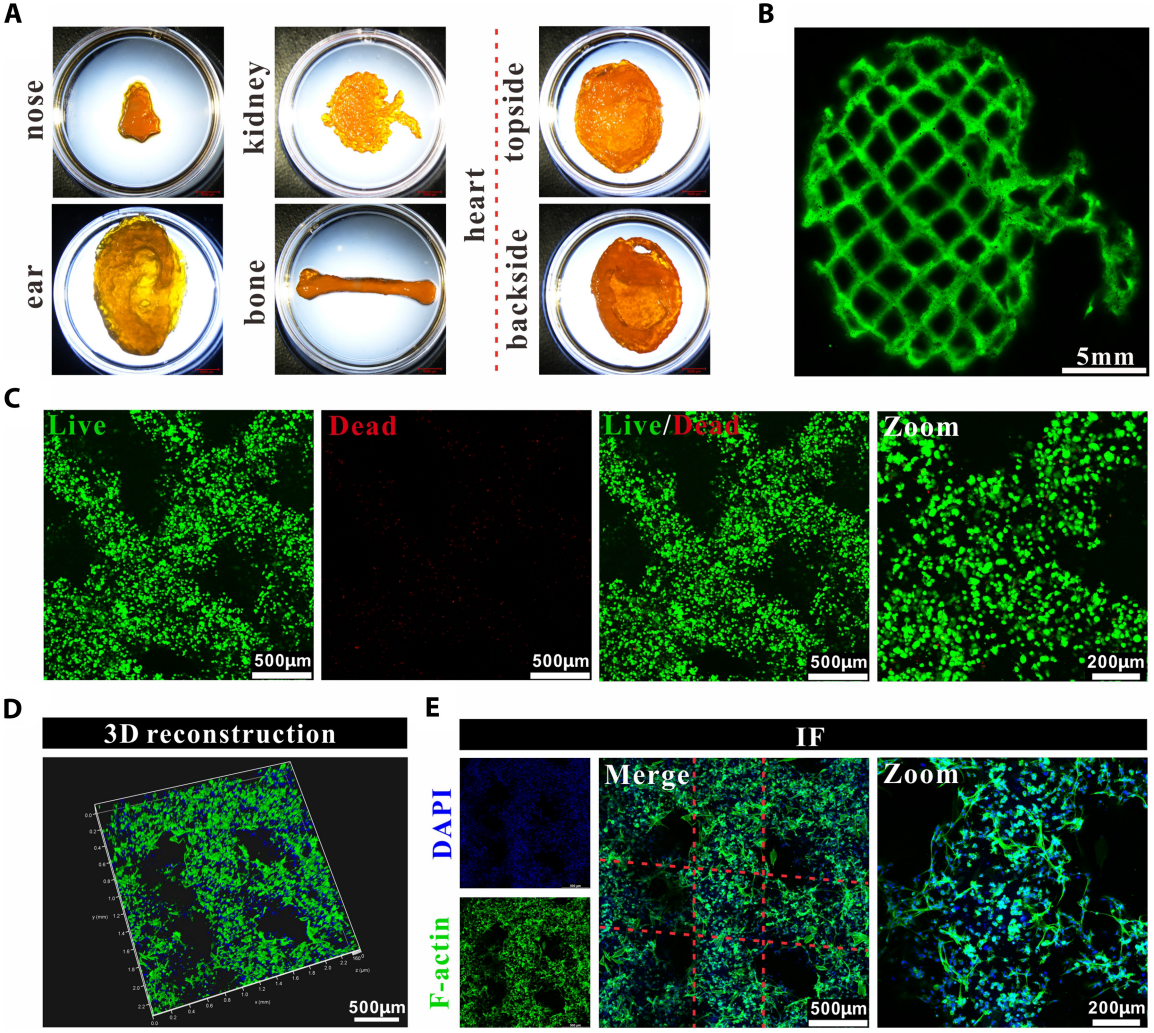
Printability of G10S6 hydrogel precursor solution. (A) The nose, ear, kidney, bone, and heart images were printed with the system. (B) Structure of each printed layer of the kidney. The structure was stained with calcein for easy observation. (C) Live and dead assay. SMCs were encapsulated in the G10S6 hydrogel for 24 h. (D) 3D reconstruction and (E) immunofluorescent (IF) staining. Cells were stained with FITC-phalloidin to display the morphology after cells were encapsulated in hydrogels for 48 h.

The muscle biomimetic scaffold was obtained using 3D bioprinting technology after the bioprinting properties of the G10S6 hydrogel precursor solution were evaluated. With the help of a supporting bath, the organs like the nose, ear, kidney, bone, and heart were printed to mimic their normal shapes (Fig. 4A). The microscopic structures of biomimetic organs are presented in Fig. 4B. The printed lines were very smooth, suggesting that the G10S6 hydrogel precursor solution could be printed in our homemade system. The specific bioprinting procedure is provided in Fig. S6.

The survival and spreading behaviors of SMCs in the scaffolds were examined. The bioinks were prepared by homogeneously mixing SMCs and G10S6 hydrogel precursor solution with a 2 × 10^7^ cells/ml cell concentration. The printed SMC-laden scaffolds were exposed to UV light (405 nm) for cross-linking. The cells were stained by calcein-AM/PI staining kit. The SMCs within the scaffold exhibited an exceptionally high rate of viability (Fig. 4C), suggesting that the printing method and the system were optimized. Additionally, the encapsulated cells were not damaged under UV light exposure. SMCs displayed extended abundant filamentous pseudopods after the SMC-laden scaffolds were cultured in DMEM complete medium for 48 h. These results suggested that hydrogel G10S6 provided SMCs with sufficient adhesion sites and space to support cell growth and spreading (Fig. 4D and E).

### 3D-printed scaffold for SMC orientation and self-organization

**Fig. 5.**
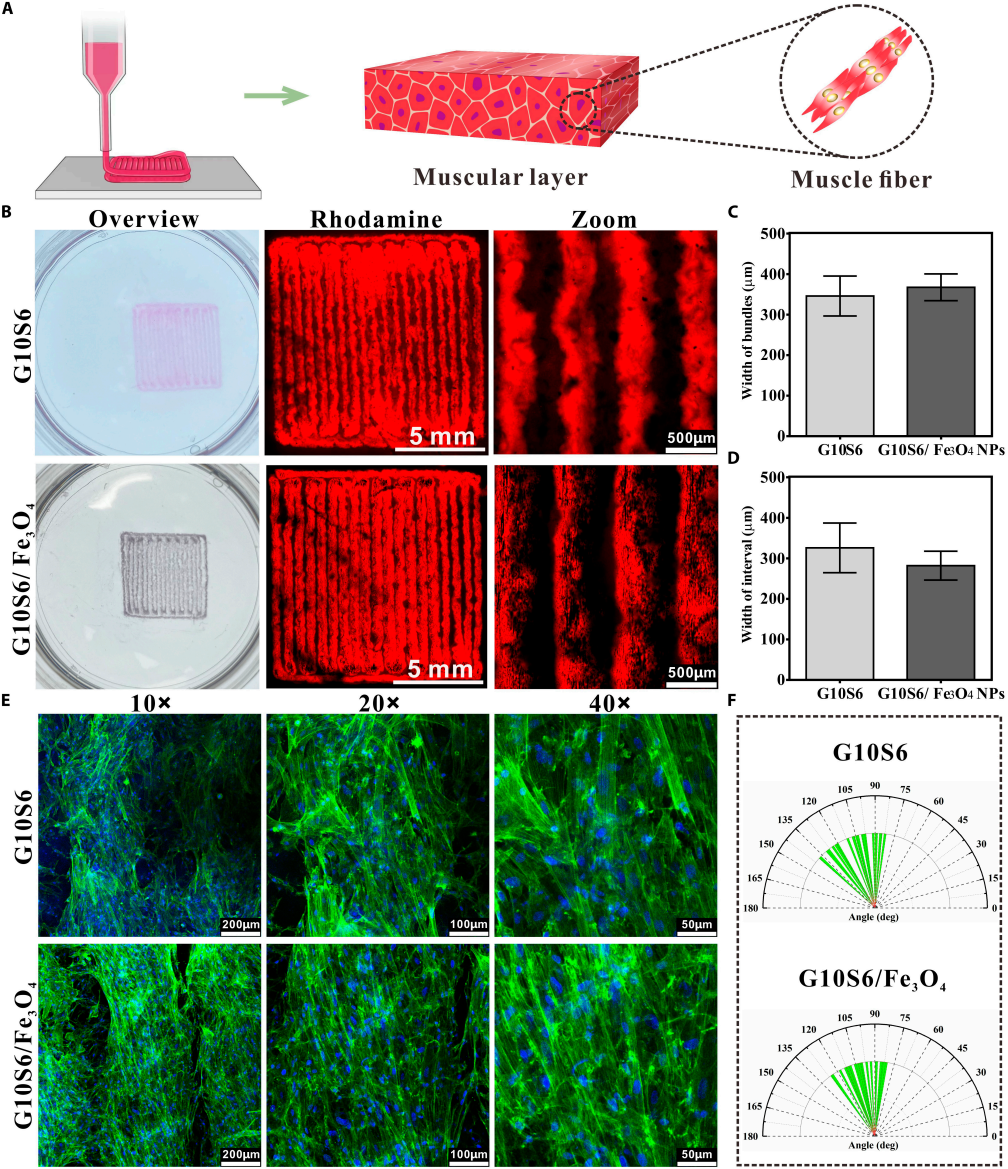
3D-printed scaffold for SMC orientation and self-organization. (A) Schematic diagram of 3D printing for aligned smooth muscle fabrication. (B) Images of the scaffold with/without Fe_3_O_4_ NPs. The scaffold was stained with rhodamine for easy observation. (C) Width of the printed streaks. (D) Interval width. (E) IF staining. Cells were stained with FITC-phalloidin to display the morphology after cells were encapsulated in hydrogels for 9 d. (F) Orientation statistics of SMCs encapsulated in hydrogel for 9 d.

Muscles are composed of muscle bundles, which are formed by muscle fibers that are closely and directionally arranged. To achieve this physiological structure, we designed a muscle scaffold, as displayed in Fig. 5A, and stacked these scaffolds using a suspension bath to promote SMC orientation and self-organization in vitro. Our previous studies have found that Fe_3_O_4_ stripes can induce cells to orient themselves in a particular direction [8]. However, previous efforts have merely confirmed the induction of 2D cellular orientation by Fe_3_O_4_ stripes and have not explored the correlation between Fe_3_O_4_ stripes and 3D cell orientation. In this study, Fe_3_O_4_ NPs were introduced into bioink and transformed into stripes under a magnetic field, acting as topographical and biochemical cues to guide cellular orientation and growth. The introduction of stripes formed by Fe_3_O_4_ NPs did not deteriorate the survival and spreading features of SMCs (Fig. S7). Then, we characterized the properties of the muscle scaffold. Figure 5B illustrates the scaffold images with/without Fe_3_O_4_ NPs. The size analyses revealed that the scaffold with Fe_3_O_4_ stripes exhibited bundles with a width of 367.4 ± 32.95 μm (Fig. 5C) and an interval of 282.0 ± 35.58 μm (Fig. 5D) between the bundles. These 2 parameters did not differ significantly from those of the control scaffold. Our findings indicated that introducing Fe_3_O_4_ stripes did not change the morphology of the muscle scaffold. Next, we observed the behavior of SMCs in these 2 scaffolds. The results found that SMCs could extend pseudopodia rapidly in both scaffolds; however, the introduction of Fe_3_O_4_ stripes improved the orientation capability of SMCs (Fig. S8). On day 9, we successfully constructed aligned smooth muscle in vitro. The scaffold with Fe_3_O_4_ stripes was more conducive to forming regular muscle bundles than the blank scaffold. The cells in the scaffold with Fe_3_O_4_ stripes were tightly and directionally arranged, while the cells tended to be disordered in the blank scaffold (Fig. 5E and F). Cells rely on pseudopodia to sense their surrounding environment and mount responses [34,35]. Fe_3_O_4_ stripes provided sufficient adhesive sites for cells, encouraging the cells to extend along the Fe_3_O_4_ stripes, thereby achieving orientation in a 3D manner.

### Fabrication of transplantable aligned smooth muscle

**Fig. 6.**
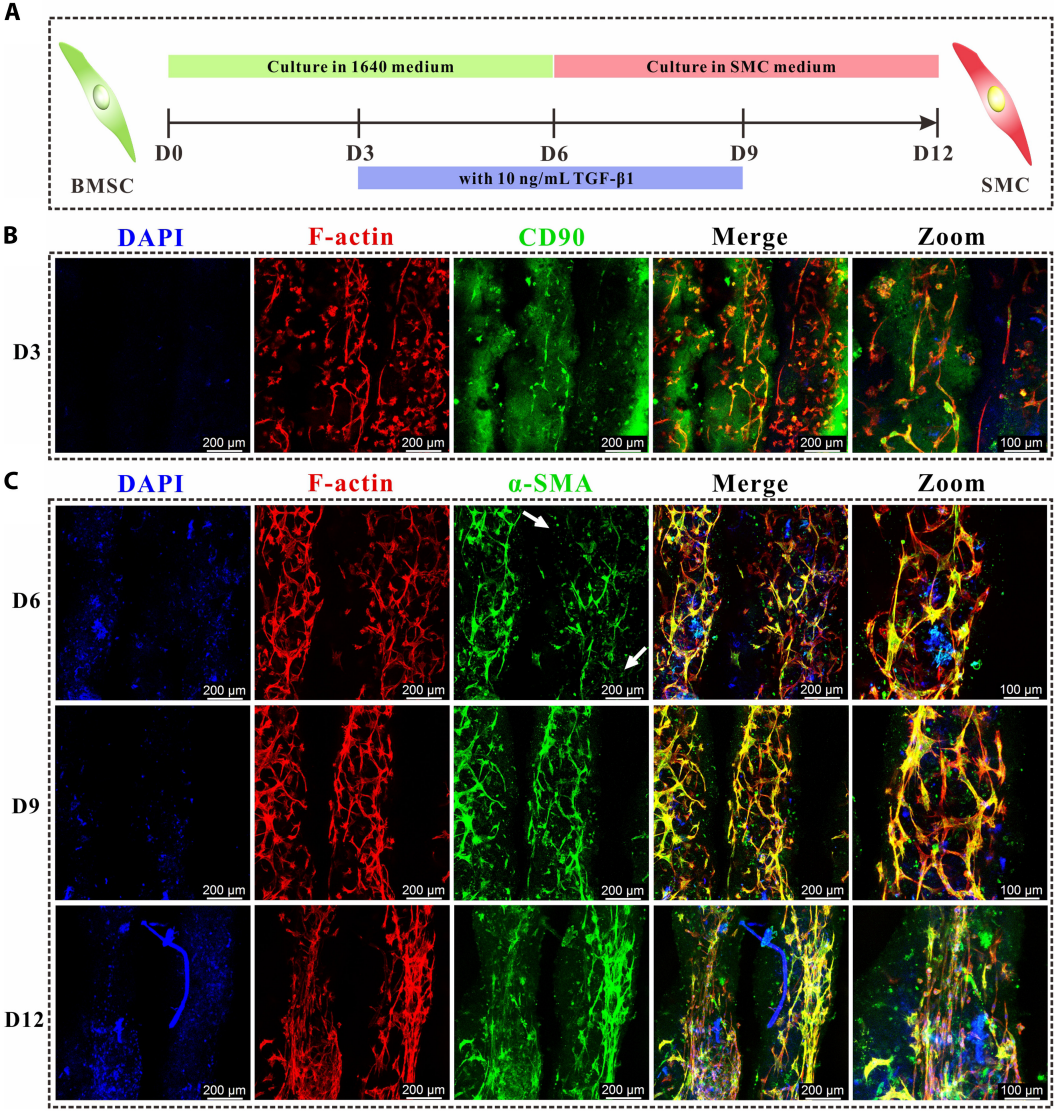
Fabrication of transplantable aligned smooth muscle. (A) Schematic diagram depicting the process of BMSC differentiation. (B) Cellular morphology and CD90 expression (a positive marker of BMSCs) were evaluated in BMSCs after cultivation for 3 d within the muscle scaffold. (C) Alterations in cellular morphology and α-SMA expression of BMSCs over time. White arrows indicate a low level of α-SMA expression.

Although we successfully constructed a muscular layer in vitro using the primary SMCs, these muscles endured immune rejection when they replaced the allogenic diseased muscles. BMSCs themselves have extremely low immunogenicity and can exert immunomodulatory effects. Thus, BMSCs were adopted for bioprinting instead of SMCs. BMSCs were extracted and cultured from rabbit bone marrow. Their identification and differentiation ability were validated (Fig. S9). With the help of TGF-β1, we successfully induced the BMSCs in the 3D scaffold to differentiate into SMCs in vitro, as displayed in Fig. 6A. On day 3, BMSCs in the scaffold exhibited limited spreading behavior and greatly expressed CD90, a positive marker for BMSCs (Fig. 6B). After TGF-β1 induction, BMSCs gradually transformed into SMCs. The areas indicated by the white arrows in Fig. 6C exhibited low α-SMA expression, suggesting the incomplete differentiation of BMSCs into SMCs. At 9 d, all cells differentiated into SMCs, confirmed by colocalization with the cytoskeleton and the expression of α-SMA. Additionally, the cells gradually elongated during BMSC differentiation into SMCs. Until 12 d, they aligned parallelly to form bundles (Fig. 6C). These results demonstrated the feasibility of constructing transplantable aligned smooth muscles in vitro using BMSCs as the seeded cells.

### Fabrication of anisotropic smooth muscle

**Fig. 7.**
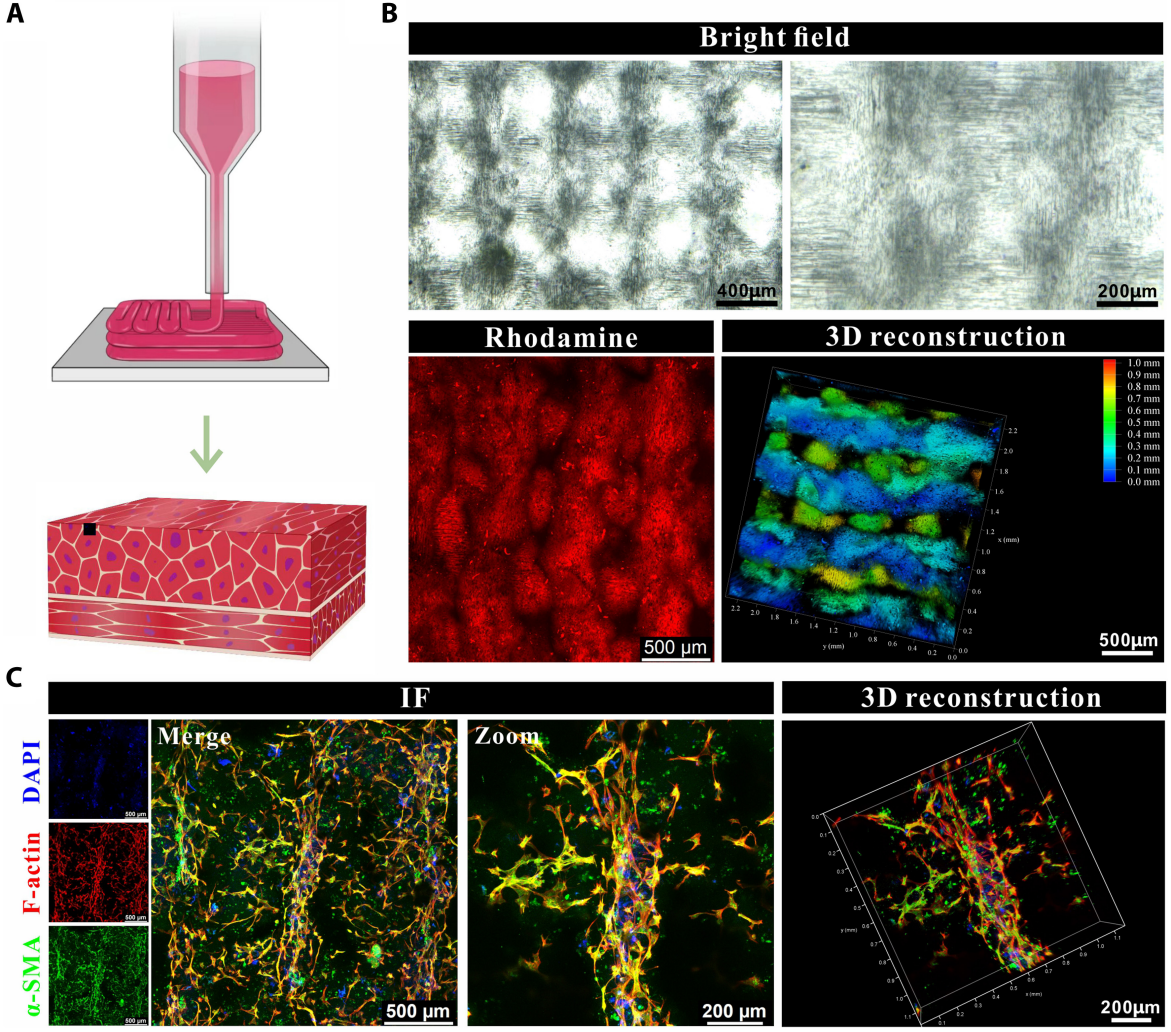
Construction of an anisotropic smooth muscle via 3D printing technology. (A) Schematic diagram of a multi-hierarchical smooth muscle fabrication. (B) Morphology of G10S6 scaffold. (C) Cell morphology. The cells were immunostained by TRITC-phalloidin and α-SMA after cultivation for 12 d within the scaffold. The expression of α-SMA in cells indicates the successful differentiation of BMSCs into SMCs.

Anisotropic aligned smooth muscle tissue orchestrates the spatiotemporal dynamics of excitation–contraction coupling, effectively modulating physiological functions. We engineered a muscle scaffold to accommodate this unique architecture, as depicted in Fig. 7A. The images of the multi-hierarchical muscle scaffold are presented in Fig. 7B. The findings revealed that the printed bundles on the upper layer of the scaffold were perpendicular to those on the lower layer, which met the requirements for mimicking the circular and longitudinal muscle layers in vitro. Additionally, we manipulated the direction of the magnetic field to ensure that the Fe_3_O_4_ stripes remained parallel to the long axis of the printed bundles. BMSCs were loaded onto the newly designed muscle scaffold and cultured for SMC differentiation. By day 9, all cells robustly expressed α-SMA and were oriented along the long axis of the printed bundles, as displayed in Fig. 7C. These results indicated the successful fabrication of a biomimetic anisotropic muscle in vitro that accommodated the specialized architecture of muscle in the body.

### Engineered muscle-promoted esophageal wound healing

The biocompatibility and degradation of the hydrogel G10S6 containing Fe_3_O_4_ NPs were evaluated by injecting G10S6 hydrogel precursor solution (200 μl) containing Fe_3_O_4_ NPs (0.2%, w/v) into rabbits and crosslinked using UV light for 2 min. The results showed that by day 3 after implantation, there were no abnormal signs such as redness, swelling, or ulceration in the corresponding skin tissues. Histopathological analysis showed a significant deposition of Fe_3_O_4_ NPs within the hydrogel, with minimal cellular infiltration. This suggests that the Fe_3_O_4_ NP-containing hydrogel G10S6 possesses good biocompatibility. By day 7 after implantation, the surrounding matrix began to gradually integrate with the hydrogel, and Fe_3_O_4_ NPs started to be phagocytosed by cells and encapsulated by the matrix, forming clumps. This phenomenon persisted until the end of the observation period, day 21. Over time, the clumps of Fe_3_O_4_ decreased, and the subcutaneous black spots further reduced in size, where Fe_3_O_4_ can be gradually metabolized by the body (Fig. S10).

**Fig. 8.**
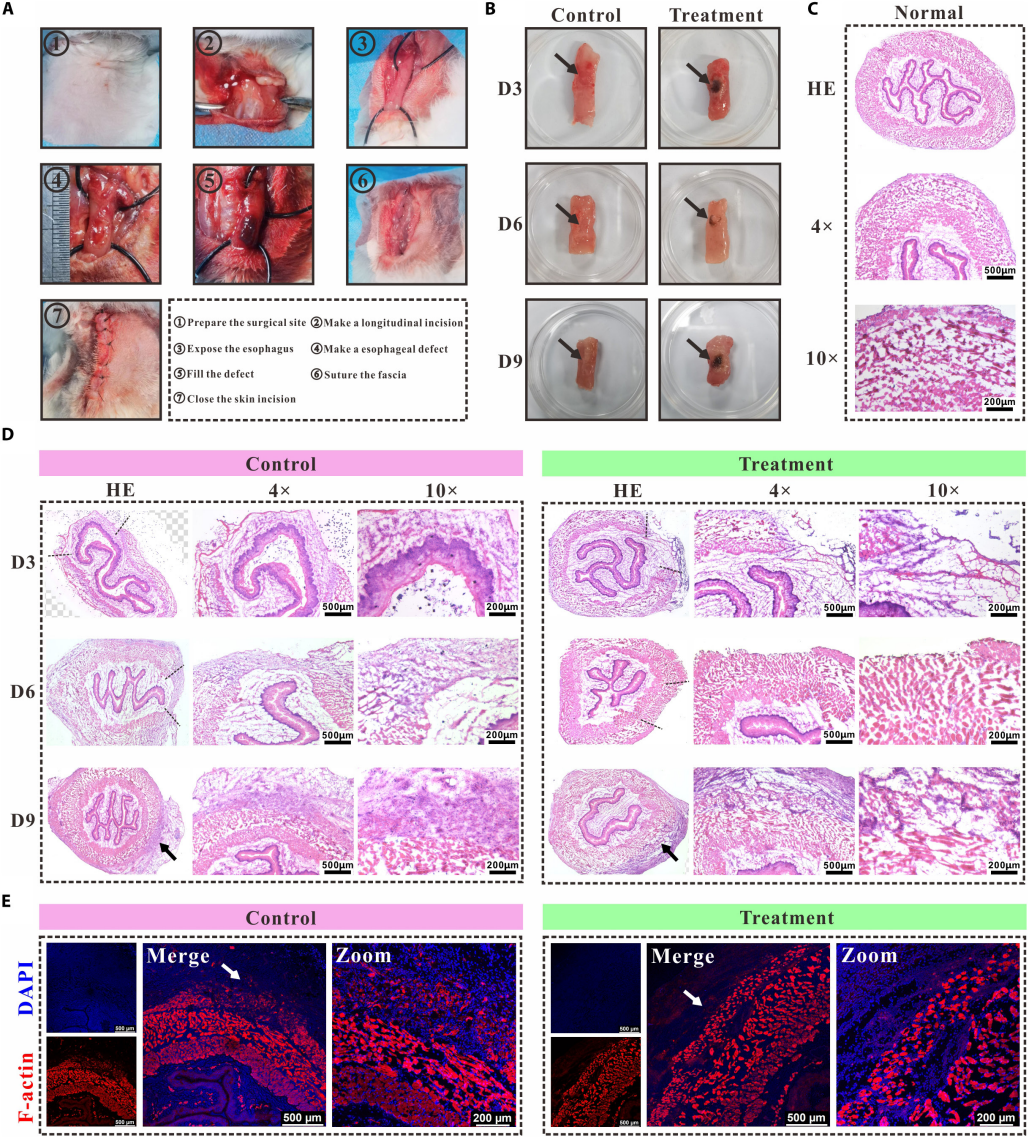
Transplantation of biomimetic muscle to repair the esophageal wound in a rabbit model. (A) Surgical procedure. The biomimetic muscle was transplanted into the esophageal wound. (B) Wound appearance of the treated and control groups at different time points. Black arrows indicate the esophageal wound. (C) H&E staining of the normal esophagus. (D) H&E staining of the muscle tissue at 3, 6, and 9 d after transplantation. The dotted lines indicate the wound size, and black arrows indicate the wound site. (E) F-actin staining of the muscle tissue in the treatment and control groups at 9 d after operation. White arrows indicate cell alignment in the outermost layer of muscle.

Animal tests were conducted to ascertain the efficacy of in vitro-engineered biomimetic muscle in replacing pathological muscle tissues. The rabbit model of esophageal muscle defects was first established. The defective muscle was replaced with the engineered 3D smooth muscle, while the control animal was left untreated. The procedural details of the animal surgeries are displayed in Fig. 8A. At 3, 6, and 9 d after operation, the animals were euthanized. Furthermore, tissue samples from the defective esophageal sites were collected for analysis. Visual assessment on day 3 revealed the integration of the biomimetic muscle with that of the esophageal muscle in the treatment group, while the control tissue exhibited little muscular deficits. As time passed, the muscle regenerated, and the defects were gradually repaired. On day 9, the esophageal defects in both groups appeared to have healed. The black dot manifested in the treatment group was due to the incomplete metabolism of Fe_3_O_4_, as displayed in Fig. 8B (black arrow).

Further, we subjected the damaged muscles to H&E staining to observe localized histopathological changes. Prior to this, the anatomical structure of the normal rabbit esophageal muscle was characterized, from the innermost to the outermost, the longitudinal, circular, and longitudinal muscles (Fig. 8C). In establishing the esophageal muscle layer defect model, the full thickness of the muscle was transected. As depicted in Fig. 8D, on day 3 after operation, both groups exhibited notable defects in the esophageal structure; however, there was a considerable cover of the lesion with a smaller wound perimeter in the treatment group compared to the control group. By day 6, the lesion in the treatment group was already sealed, while the defect in the control group was merely reduced. By day 9, the defect in the treatment group was completely healed, with the biomimetic muscle fully integrated with the regenerating native muscle tissue; the muscle defect in the control group was also closed at this stage. We employed TRITC-phalloidin to highlight the F-actin in SMCs and determine the integrity of muscular layer repair. The results found that the esophageal muscle layer in the treatment group was essentially recovered, whereas the repair in the control group was incomplete as the cell alignment in the outermost layer of muscle was not as compact (Fig. 8E).

**Fig. 9.**
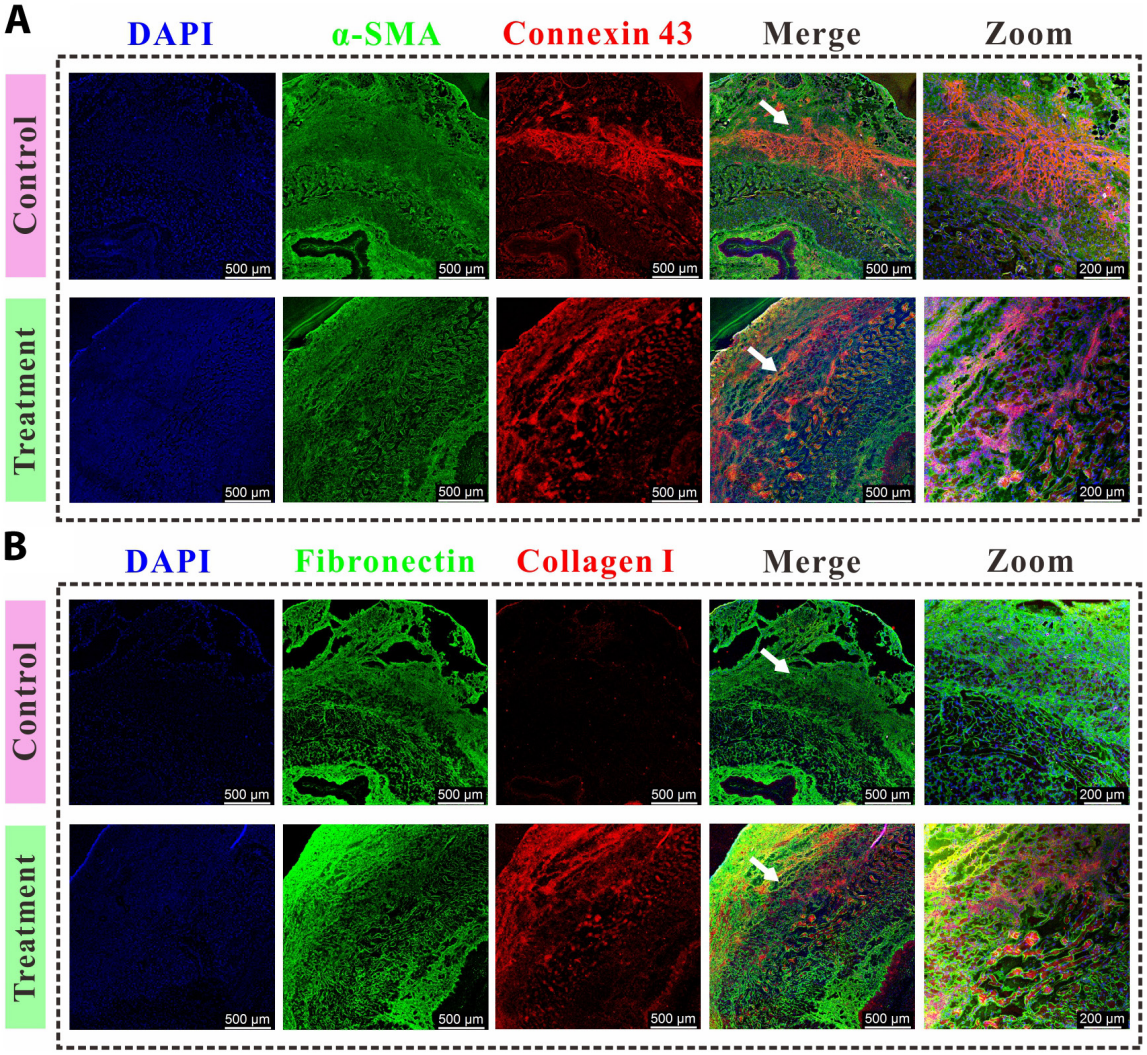
Regeneration of esophageal smooth muscles after biomimetic muscle transplantation. The tissue was immunostained with α-SMA, connexin 43 (A), and fibronectin, collagen I (B) at day 9 after transplantation of biomimetic muscle. The tissue without treatment was used as the control.

On day 9, IF staining was performed to investigate the expression of α-SMA, connexin 43, fibronectin, and collagen I at the muscle injury site. α-SMA is a marker protein specific to SMCs, whereas connexin 43 acts as the principal gap junction protein within SMCs [36]. Our findings indicated the presence of α-SMA expression at the wound sites in both experimental groups, suggesting the replenishment of muscle cells and restoration of muscle functionality. Notably, connexin 43 revealed a concentrated expression at the injury sites in the control group, attributed to the less dense arrangement of muscle cells at the wound, leading to an increased secretion of gap junction proteins. This was consistent with the results obtained for F-actin staining. Conversely, in the treatment group, connexin 43 was found to be diffusely expressed, thus enhancing the intercellular connectivity within muscular tissue (Fig. 9A). Fibronectin and collagen I, associated with the SMCs ECM [37,38], were well expressed at the injury sites within the treatment group, in contrast to the control group, where lower expression levels were observed. This suggested a more effective reconstruction of the ECM in the injured muscle of the treatment group compared to its counterpart in the control group (Fig. 9B).

## Discussion and Conclusion

This study successfully demonstrated the in vitro fabrication and in vivo application of biomimetic smooth muscle, highlighting the potential of magnetic induction and bioprinting technologies in constructing biomimetic tissues. This achievement is underpinned by the desirable properties of the hydrogel G10S6, which include a porous microstructure, mechanical stability, degradation, and cell-adaptive network dynamics.

In this work, we developed a muscle-mimetic Gel/silk fibroin hydrogel scaffold containing Fe_3_O_4_ NPs using the 3D bioprinting technology combined with magnetic induction. The hydrogel scaffold can be designed to fabricate aligned or anisotropic muscles to accommodate special architectures of muscular tissues in the body. The Fe_3_O_4_ NPs provide micro/nanoscale geometric stripes under the induction of linear magnetic force, which directs the oriental growth of SMCs and promotes the differentiation of BMSCs into SMCs and the in vitro self-organization of muscle tissue.

The hierarchical structure of human tissues/organs is highly diversified and complex. High-orientation structures between different layers are interconnected through tissue-specific ECM, forming an integration with smooth transitions in structure and properties [39]. To achieve this, a high degree of cellular alignment and a multi-level structure are required. Our study addressed this principal challenge by successfully replicating natural tissue architecture from complex molecular components to macroscopic scales.

Recently, the implantation of BMSC-derived seed cells in vivo has demonstrated the advantages of low immunogenicity and the powerful potential for repairing target tissues/organs including smooth muscle tissue [25], hepatic tissue [40], tendon [41], and cartilage [42]. This work employed a similar strategy by utilizing BMSC-derived SMCs toward the construction of biomimetic muscle in vitro, aiming to reduce immunogenicity during allogeneic transplantation.

Animal experiments have indicated that our in vitro-engineered biomimetic muscle facilitated the organized alignment of SMCs and the reconstruction of associated ECM, thereby accelerating the repair of esophageal muscular defects. Despite incorporating the inorganic material Fe_3_O_4_ within the biomimetic muscle, it did not impede the repair process. In recent years, magnetic NPs have been extensively employed for imaging, diagnosis, and therapeutic purposes [43–45]. However, to date, few studies have reported their use in conjunction with organic materials to direct the 3D alignment of cells that mimic the natural architecture of the muscle, contributing to the repairing of the defect.

Therefore, we present the work with several core innovations, for example, (a) optimizing the composition of GS hydrogel to support cell growth, proliferation, and differentiation while maintaining the appropriate stiffness necessary for sustaining the biomimetic scaffold structure; (b) employing a magnetic field to induce the self-assembly of Fe_3_O_4_ NPs within the hydrogel, using it as a geometric cue to guide cellular orientation in 3D space; (c) introducing a novel method for constructing aligned or anisotropic biomimetic muscle in vitro.

This study carries significant implications in repairing esophageal smooth muscle defects and highlights the potential applications in other tissues/organs. These innovative scaffolds could provide pivotal clinical benefits and pave the way for effective treatments of smooth muscle impairments. However, this study has certain limitations, such as the difficulty in metabolically clearing Fe_3_O_4_ in the short term and the overly complex procedures for constructing aligned or anisotropic muscle in vitro. Therefore, conducting further research to address these issues will be the focus of our future work.

In summary, we developed a novel muscle-mimetic Gel-silk fibrous hydrogel matrix embedded with aligned Fe_3_O_4,_ which self-assembles by combining 3D bioprinting and magnetic field induction for adjusting the cells to align and grow in 3D bulk. The optimized scaffold possesses desirable properties, including porous microstructure, mechanical stability, degradation, and cell-adaptive network dynamics, and also promotes the in vitro alignment growth and directional differentiation of BMSCs into SMCs, thereby forming a transplantable biomimetic muscle. In a rabbit model with muscular defects, the engineered muscle enhanced the SMCs’ alignment and restored the ECM, closely mimicking the native architecture of circular and longitudinal smooth muscle. This biomimetic muscle introduces a novel approach for repairing and replacing diseased or damaged muscle tissue.

## Data Availability

Materials from the present study are available from the corresponding author on reasonable request.
