# Supplementary material for: Fabrication of 3D Biomimetic Smooth Muscle Using Magnetic Induction and Bioprinting for Tissue Regeneration
Source: Biomater Res. 2024 Sep 9;28:0076. doi: 10.34133/bmr.0076 (PMC11382380; doi:10.34133/bmr.0076)
Supplement: Supplementary 1 — Figs. S1 to S10 [file bmr.0076.f1.docx]

**Supplemental Material**

**Fabrication of 3D biomimetic smooth muscle using magnetic induction and bioprinting for tissue regeneration**

Yang Luo^1#^, Zeming Hu^1#^, Renhao Ni^1^, Rong Xu^1^, Jianmin Zhao^2^, Peipei Feng^3^, Tong Zhu^1^, Yaoqi Chen^2^, Jie Yao^4,5^, Yudong Yao^1,5^, Lu Yang^4*^, Hua Zhang^1,5,6*^, Yabin Zhu^1*^

**Affiliation**

^1^ Health Science Center, Ningbo University, Ningbo, 315211, China

^2^ Sir Run Run Shaw Hospital, Zhejiang University School of Medicine, Hangzhou, 310016, China

^3^ Ningbo Institute of Innovation for Combined Medicine and Engineering, The Affiliated Lihuili Hospital of Ningbo University, Ningbo, 315046, China

^4^ The First Affiliated Hospital of Ningbo University, Ningbo, 315010, China

^5^ Research Institute of Smart Medicine and Biological Engineering, Ningbo University, Ningbo, 315211, China

^6^ State Key Laboratory of Molecular Engineering of Polymers, Fudan University, Shanghai, 200438, China

^#^ These authors contributed equally to this work.

*Corresponding authors: [zhuyabin@nbu.edu.cn](mailto:zhuyabin@nbu.edu.cn); zhanghua@nbu.edu.cn; [yanglu@nbu.edu.cn](mailto:yanglu@nbu.edu.cn).


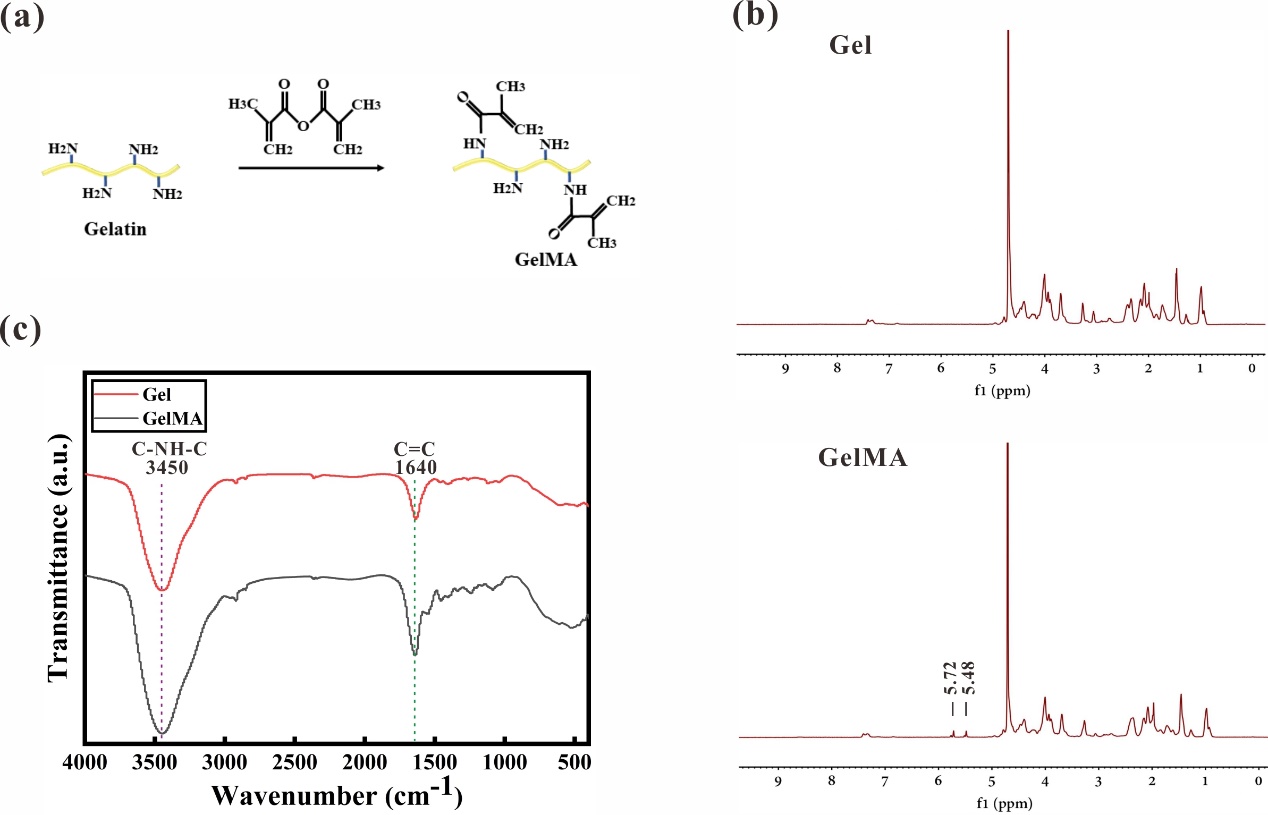


Figure S1. Synthesis and characterization of GelMA. (a) The synthetic route of GelMA; (b) ^1^H-NMR spectra and (c) ATR-FTIR spectra of Gel and GelMA.


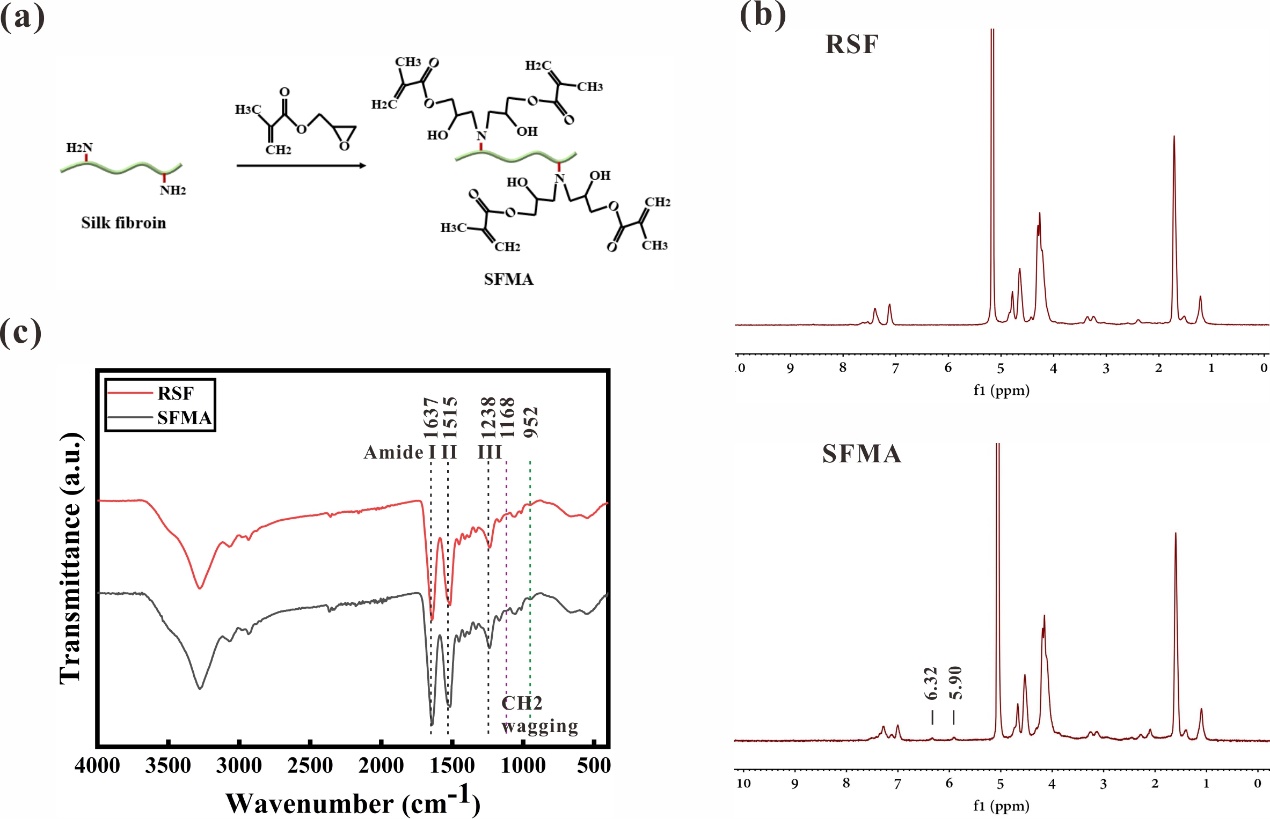


Figure S2. Synthesis and characterization of SFMA. (a) The synthetic route of SFMA; (b) ^1^H-NMR spectra and (c) ATR-FTIR spectra of RSF and SFMA.


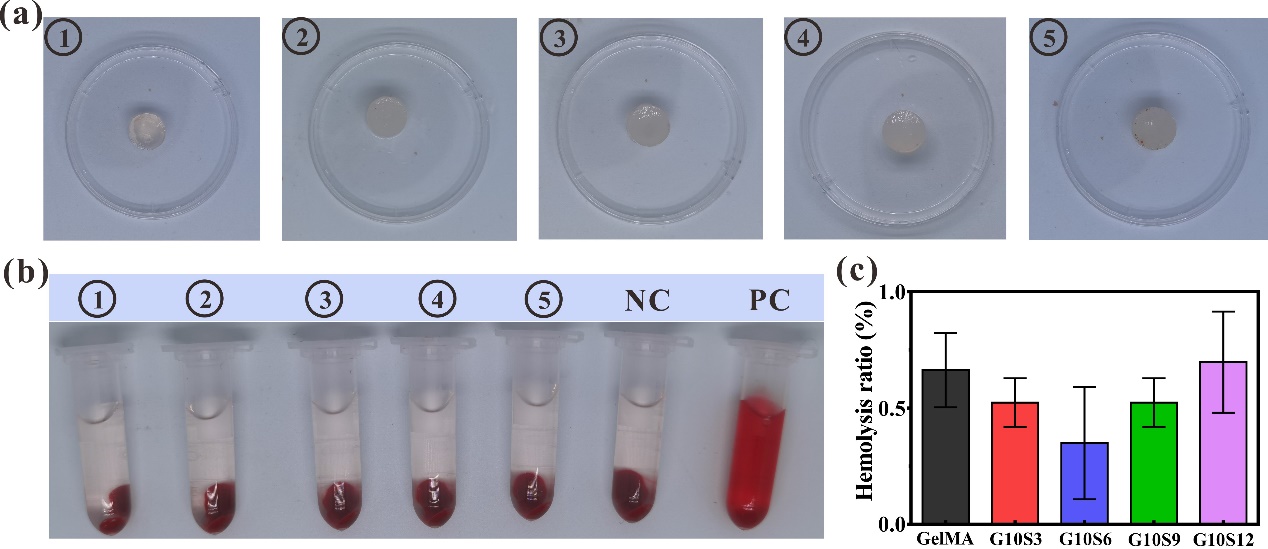


Figure S3. Blood compatibility of GS hydrogel. (a) Blood cells adhesion; (b & c) Hemolysis rate upon incubation with GelMA (1), G10S3 (2), G10S6 (3), G10S9 (4), G10S12 (5), NC (negative control) and PC (positive control).


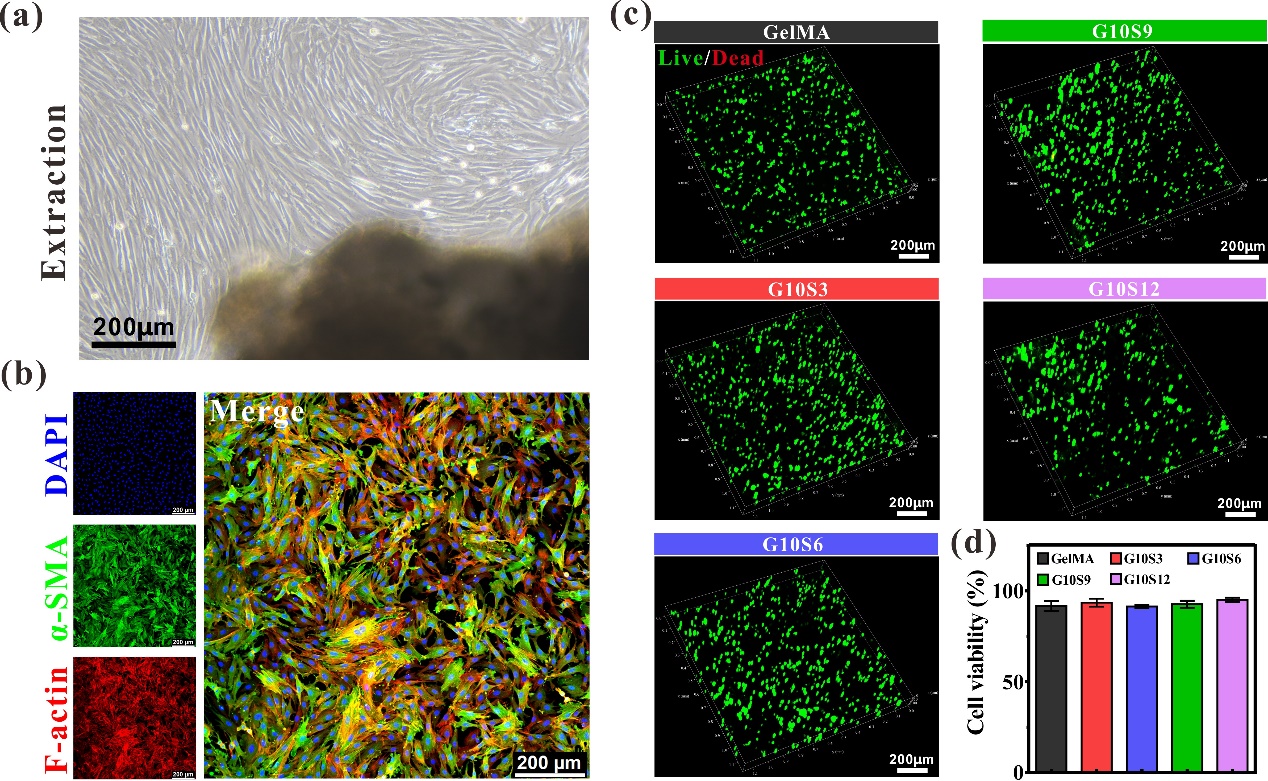


Figure S4. Cytocompatibility of GS hydrogel. (a) Extraction and (b) identification of SMCs; (c & d) Live/Dead assay; cells were encapsulated for 24 h.


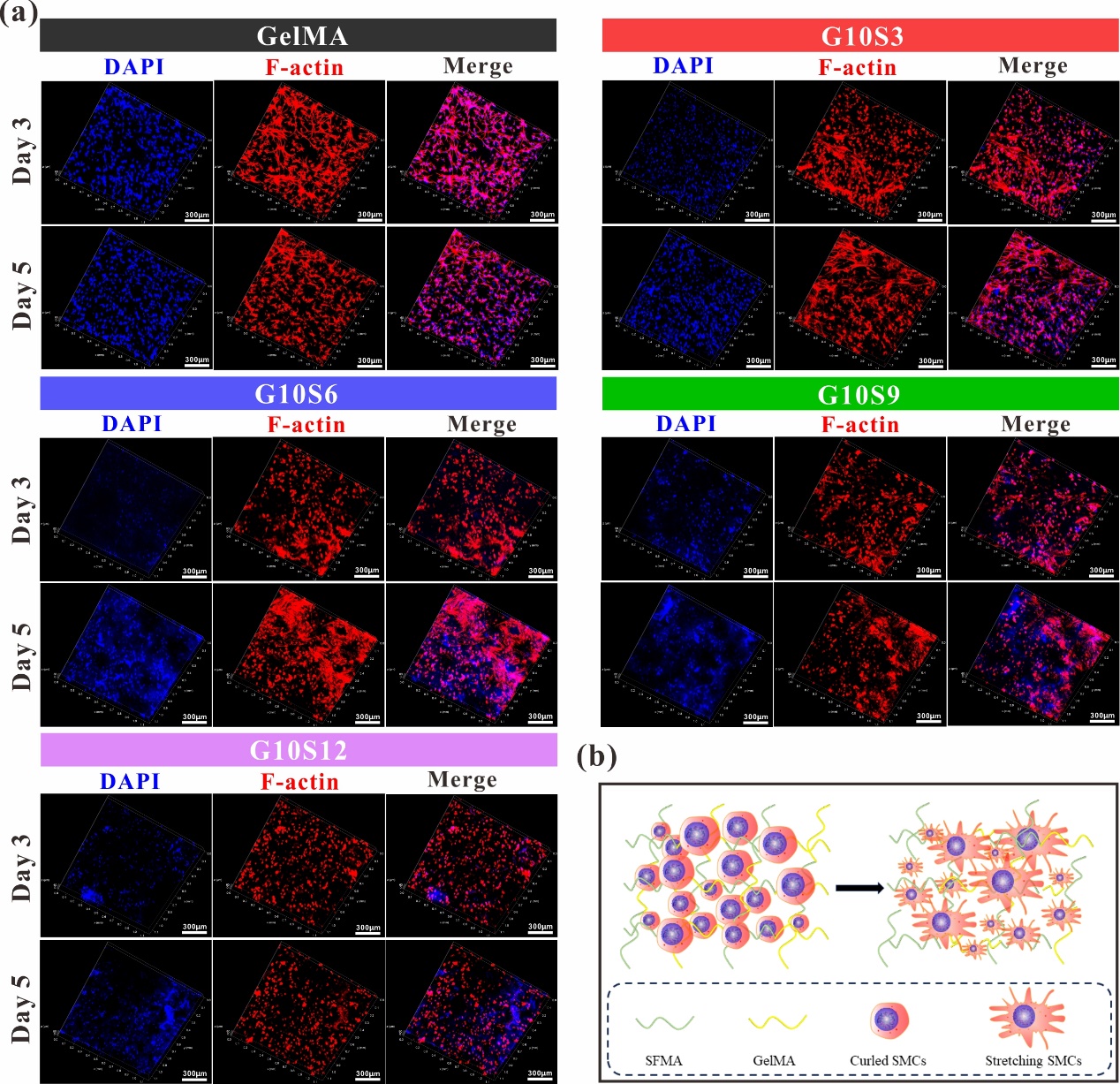


Figure S5. SMCs behavior in GS hydrogel. (a) Images of cell morphology after cell encapsulated in hydrogels for 3 d and 5 d; (b) Schematic diagram of cells stretch in hydrogels.


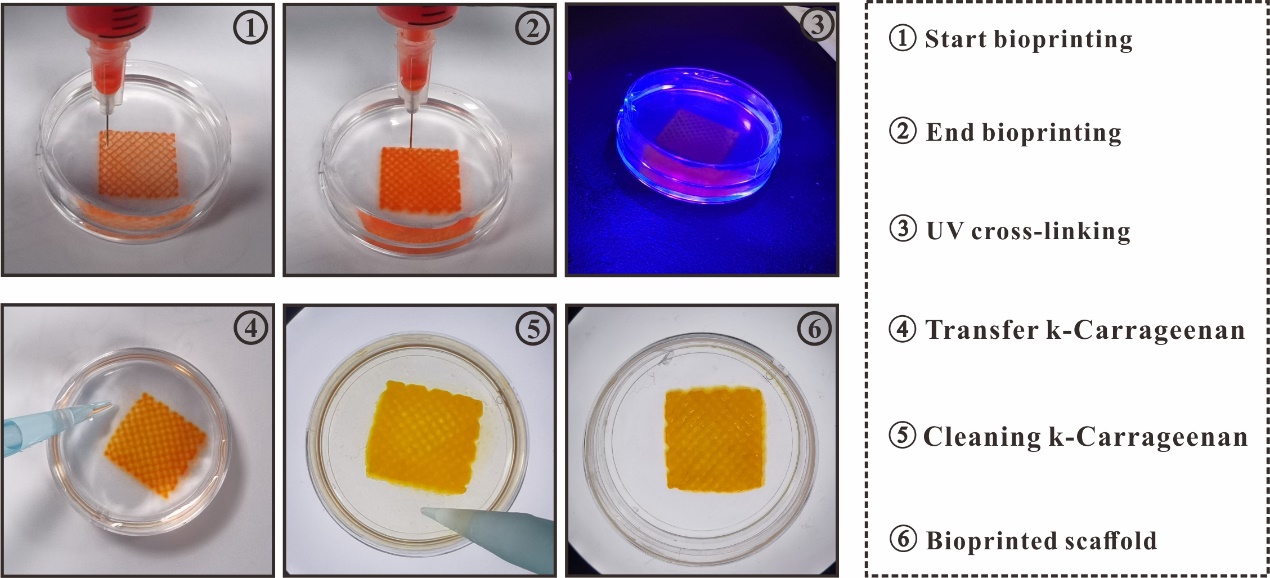


Figure S6. The bioprinting process for generating scaffolds.


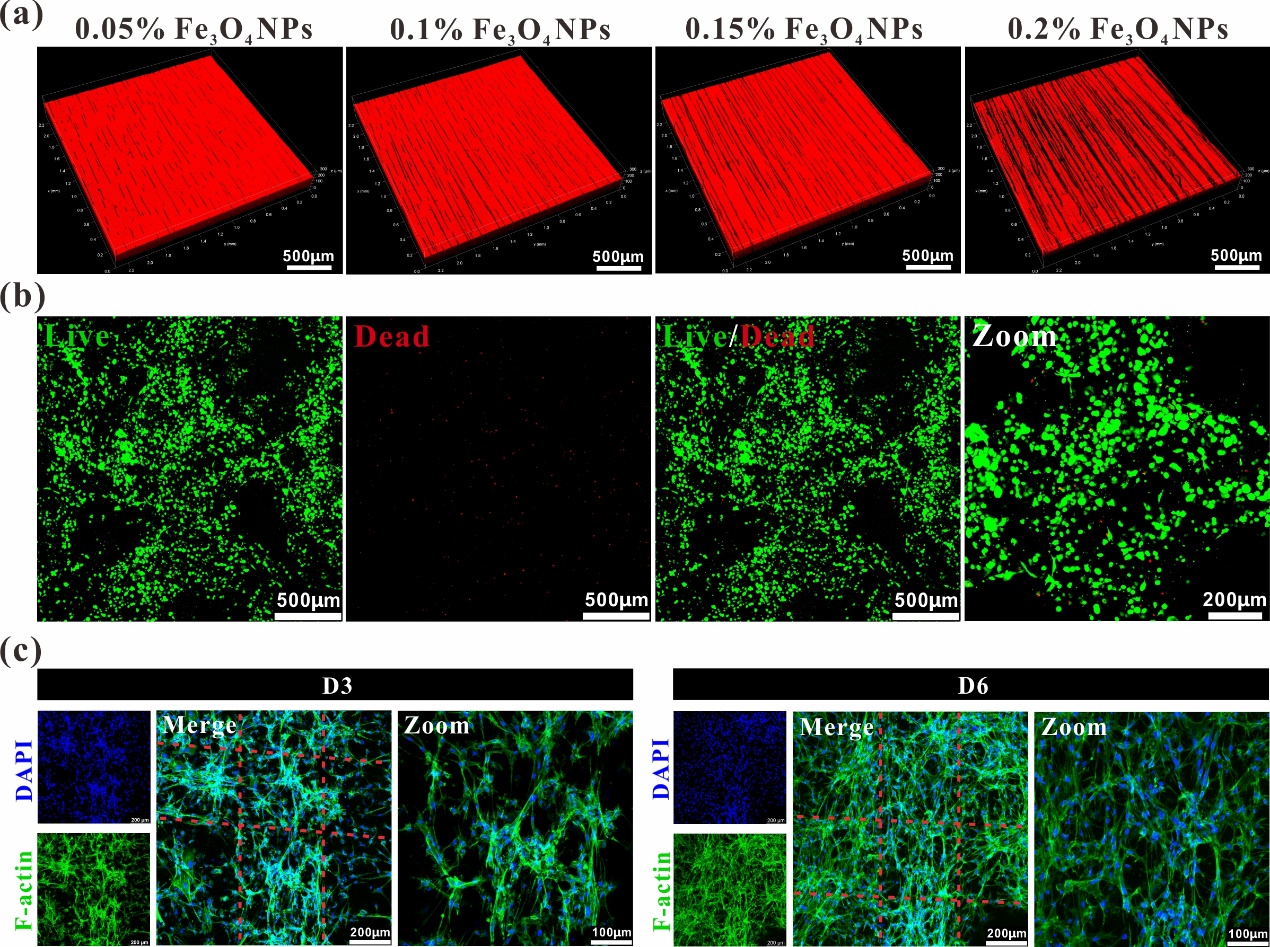


Figure S7. Cytocompatibility of Fe_3_O_4_ stripes in scaffolds. (a) Images of Fe_3_O_4_ stripes formed with different concentrations of Fe_3_O_4_ NPs in hydrogels (hydrogels in red and Fe_3_O_4_ stripes in black); (b) Live and dead assay; cell viability of encapsulated SMCs for 24 h; 0.2%Fe_3_O_4_ NPs were introduced into bioink and transformed to stripes under a magnetic field (c) Images of cell morphology after cell encapsulated in scaffolds for 3 d and 6 d.


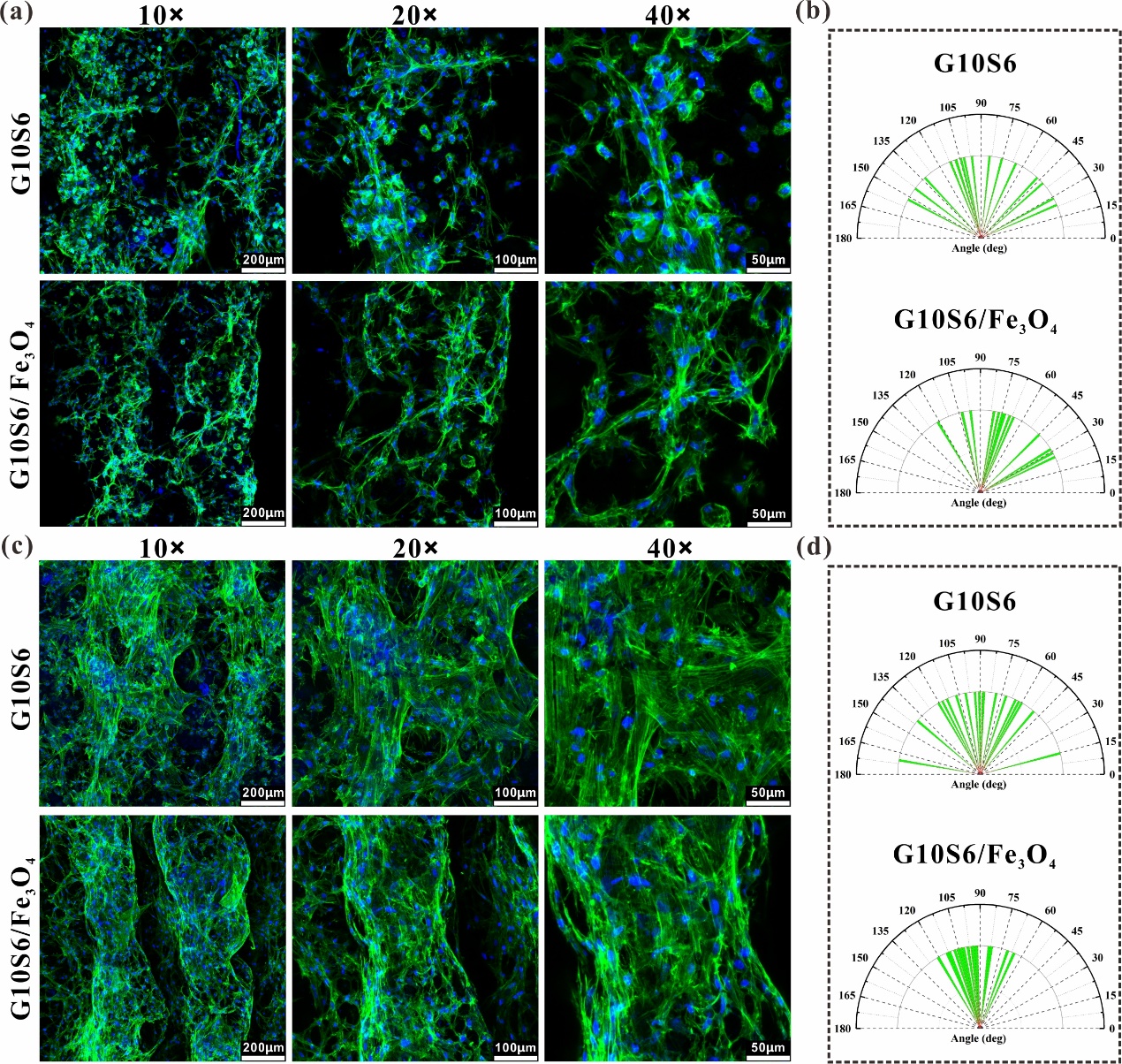


Figure S8. The process for generating smooth muscle bundles via bioprinting technology. (a) IF images and (b) orientation statistics of SMCs in muscle scaffold for 3 d; (c) IF images and (d) orientation statistics of SMCs in muscle scaffold for 6 d.


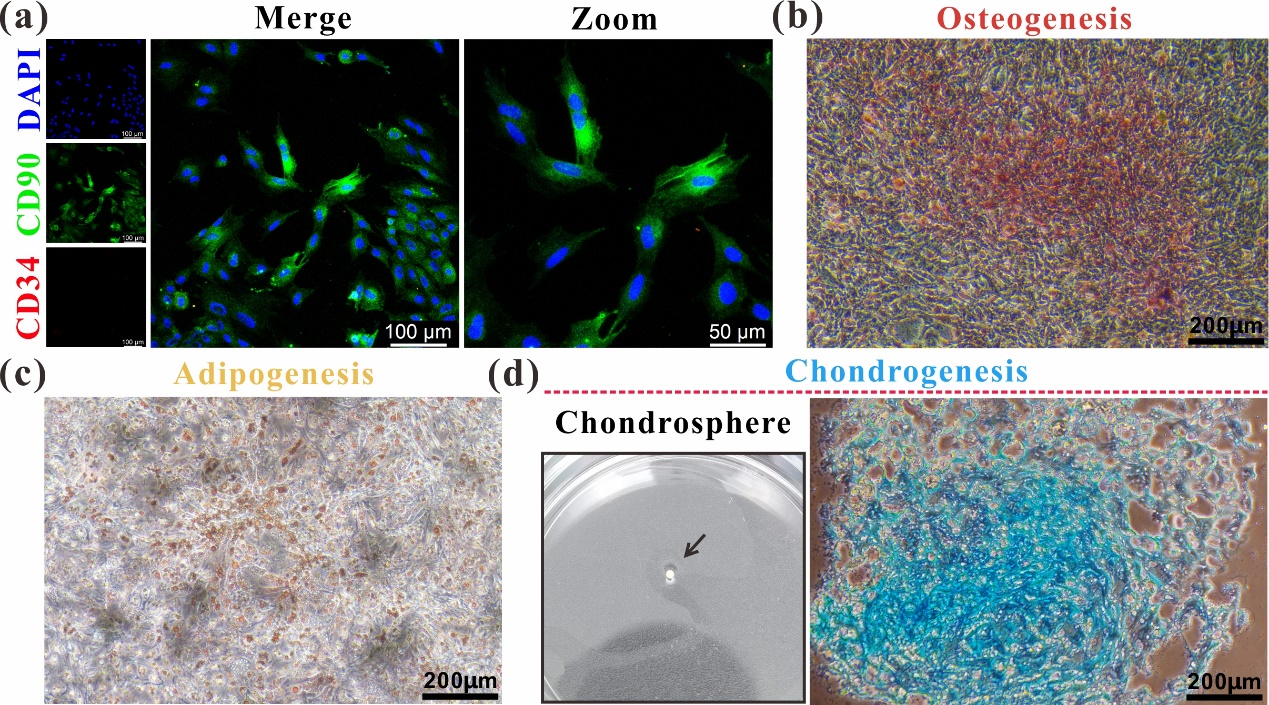


Figure S9. Identification and differentiation capacities of BMSCs. (a) IF technique for the identification of BMSCs (CD90 as a positive marker, CD34 as a negative marker); Confirmation of BMSC potential for differentiation into (b) osteoblasts, (c) adipocytes and (d) chondrocytes.


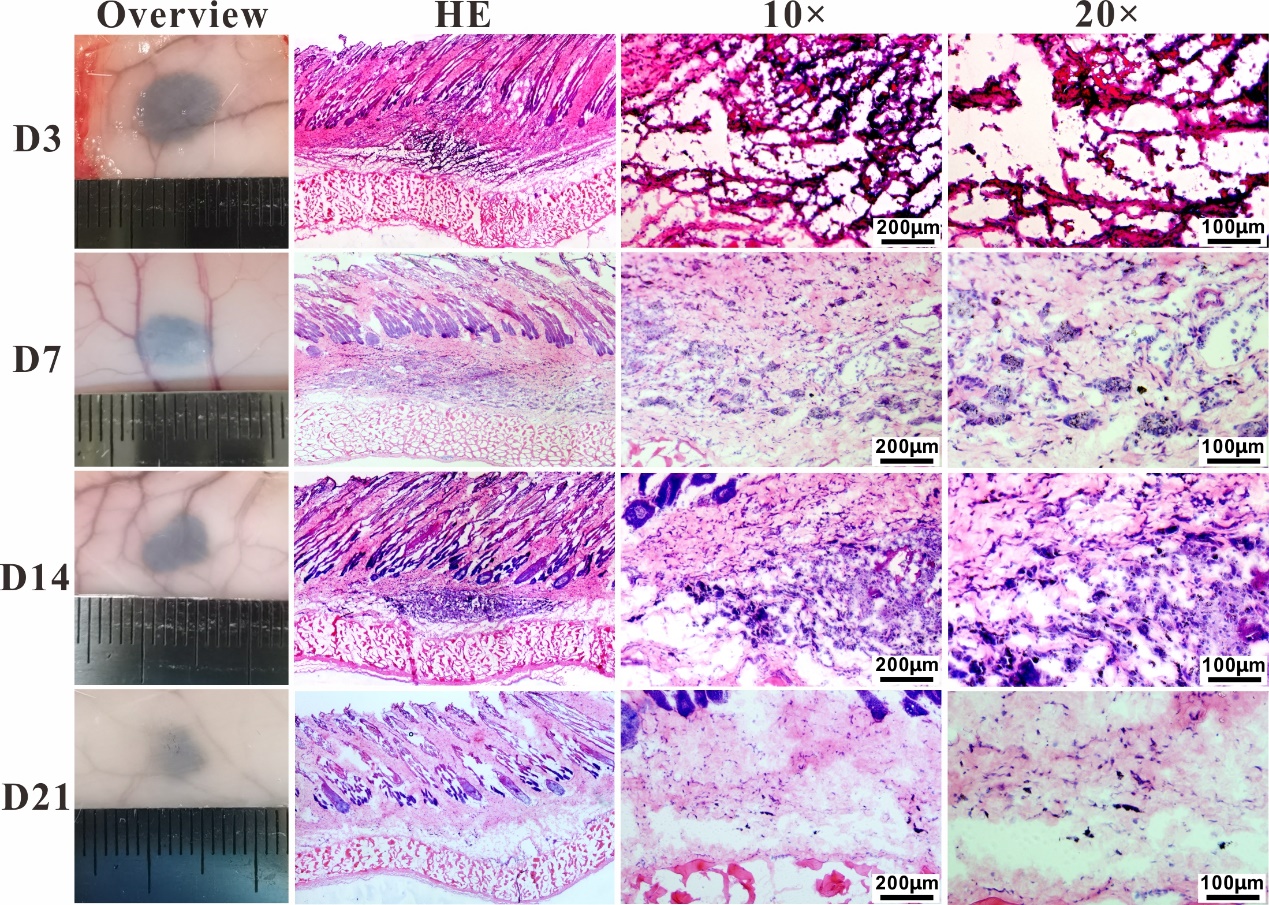


Figure S10. Biocompatibility and degradation of hydrogel G10S6 containing Fe_3_O_4_ NPs. G10S6 hydrogel precursor solution (200 μL) containing Fe_3_O_4_ NPs (0.2%, w/v) was injected subcutaneously into rabbits and crosslinked using UV light for 2 min. The corresponding skin tissue was collected for H&E staining at 3, 7, 14 and 21 d after implantation.
